# Tumor-derived annexin A1 drives lenvatinib resistance by regulating SOX2/STAT3/S100A6 feedback loop and M2 macrophage polarization in hepatocellular carcinoma

**DOI:** 10.1038/s41419-026-09118-w

**Published:** 2026-07-29

**Authors:** Catherine Yu Jia Gu, Carmen Oi Ning Leung, Rainbow Wing Hei Leung, Wing Ki Chau, Martina Mang Leng Lei, Yi Tian Chen, Sze Man Chan, Gregory Kenneth Muliawan, Yuan Gao, Qian Qian Yu, Ming Liu, Xiao Qi Wu, Jin Ding, Xin Wang, Stephanie Ma, Terence Kin Wah Lee

**Affiliations:** 1 Department of Applied Biology and Chemical Technology, The Hong Kong Polytechnic University, Hong Kong, China; 2 State Key Laboratory of Cancer Biology, Biotechnology Center, School of Pharmacy, Fourth Military Medical University, Xi’an, China; 3 Department of Oncology, Tongji Hospital, Tongji Medical College, Huazhong University of Science and Technology, Wuhan, China; 4 Affiliated Cancer Hospital Institute of Guangzhou Medical University, Guangzhou Municipal and Guangdong Provincial Key Laboratory of Protein Modification and Degradation, School of Basic Medical Sciences, Guangzhou Medical University, Guangzhou, China; 5 State Key Laboratory of Respiratory Disease, Guangzhou Medical University, Guangzhou, China; 6 Xi’an Boxin Zhida Biotechnology Co. Ltd, Xi’an, China; 7 Clinical Cancer Institute, Center for Translational Medicine, Naval Medical University, Shanghai, China; 8 Department of Surgery, The Chinese University of Hong Kong, Hong Kong, China; 9 School of Biomedical Sciences, Li Ka Shing Faculty of Medicine, The University of Hong Kong, Hong Kong, China; 10 State Key Laboratory of Liver Research, The University of Hong Kong, Hong Kong, China; 11 Research Institute for Future Food, The Hong Kong Polytechnic University, Hong Kong, China

**Keywords:** Cancer stem cells, Cancer microenvironment

## Abstract

Lenvatinib has been approved by the FDA as a front-line treatment for advanced hepatocellular carcinoma (HCC), but its survival benefits are limited by acquired drug resistance. In this study, we investigate the mechanisms underlying lenvatinib resistance in HCC by establishing lenvatinib-resistant (LenR) patient-derived tumor xenograft (PDTX) and analyzing their genetic profiles via single-cell RNA sequencing (scRNA-seq). Through single-cell regulatory network inference and clustering (SCENIC) analysis, we find that Annexin A1 (*ANXA1*) is significantly enriched in LenR HCC clusters. Using knockdown and overexpression strategies, we demonstrate the critical role of ANXA1 in regulating cancer stemness-driven lenvatinib resistance. ANXA1 mediates resistance by regulating S100A6 expression, while its expression is regulated by SOX2 via promoter activation, forming a positive feedback loop with STAT3. Notably, we reveal that ANXA1 secreted from LenR HCC cells reshapes the tumor microenvironment (TME) by inducing M2 macrophage polarization via FPR2 binding and activation of ERK and NF-κB signaling, leading to decreased T cell infiltration. Targeting ANXA1 via an adeno-associated virus serotype 8 (AAV8) approach improves lenvatinib efficacy in our LenR *Tp53*^KO^/*Myc*^OE^ HCC mouse model, accompanied by the suppression of an immunosuppressive TME. In conclusion, HCC-derived ANXA1 regulates lenvatinib resistance both intrinsically and extrinsically. Targeting the SOX2/ANXA1/STAT3/S100A6 positive loop may provide a novel therapeutic strategy for HCC treatment.

**Figure:**
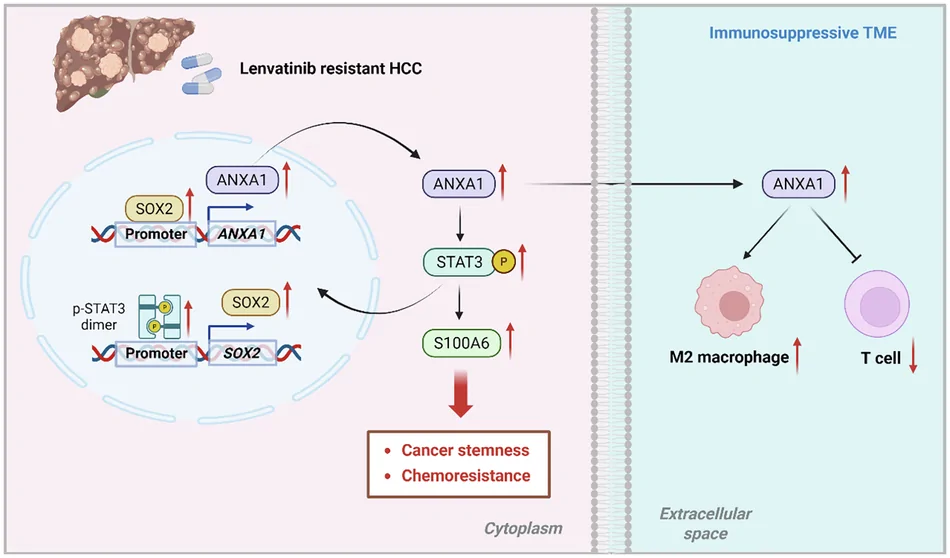

## Introduction

Hepatocellular carcinoma (HCC) is the sixth most commonly diagnosed cancer and the third leading cause of cancer-related death worldwide [1]. HCC has entered a new era with the development of molecular-targeted therapies, such as sorafenib, which improves the survival of HCC patients at advanced stages [2]. However, the survival benefit is modest. Lenvatinib, a multikinase inhibitor (MKI), is utilized as a first-line treatment and targets receptor tyrosine kinases (RTKs), including platelet-derived growth factor receptor α (PDGFRα), vascular endothelial growth factor receptors (VEGFRs) 1-3, fibroblast growth factor receptors (FGFRs) 1-4, RET, and KIT, whereas regorafenib, cabozantinib, and ramucirumab serve as second-line options [3,4]. Despite their effectiveness, these therapies provide limited survival benefits. These clinical observations suggest that suboptimal outcomes are associated with acquired drug resistance. Recent Phase II clinical trials have demonstrated that lenvatinib combined with anti-PD1 antibodies shows promising efficacy and tolerable safety profiles [5]. Therefore, a deeper understanding of the molecular mechanisms underlying lenvatinib resistance and how lenvatinib-resistant (LenR) tumors shape the immune landscape in the tumor microenvironment (TME) may pave the way for novel therapeutic strategies for HCC patients.

Researchers have investigated the molecular mechanisms of lenvatinib resistance in HCC and identified potential therapeutic targets. For instance, CRISPR/Cas9 library screening identified neurofibromin 1 (NF1) and dual-specificity phosphatase 9 (DUSP9) as key drivers of resistance [6]. NF1 or DUSP9 depletion reactivates MAPK/ERK signaling, leading to FOXO3 inactivation and degradation and, ultimately, lenvatinib resistance. Separately, phosphorylated MYH9 (Ser1943) recruits USP22 to deubiquitinate and stabilize HIF-1α, which promotes cancer stemness and further contributes to lenvatinib resistance [7]. These studies elucidated intrinsic signaling pathways regulating lenvatinib resistance; however, the remaining gap is how resistant cells remodel the TME. To address this question, we first established LenR PDTX in NOD/SCID mice by administering four rounds of lenvatinib [8] and identified the critical molecules/pathways involved in acquired drug resistance at the single-cell level via single-cell RNA sequencing (scRNA-seq) analysis. Upon analysis, several liver cancer stem cell markers were found to be upregulated in LenR HCC. Based on SCENIC (single-cell regulatory network inference), we found that Annexin A1 (*ANXA1*) was significantly enriched in LenR HCC clusters. ANXA1 is a calcium-dependent phospholipid-binding protein that belongs to the annexin superfamily [9]. Increasing evidence shows that elevated ANXA1 expression is linked to poor prognosis and reduced overall survival in patients with breast cancer, melanoma, colorectal cancer, prostate cancer, and lung adenocarcinoma [10–14]. Although the role of ANXA1 in cancer development and progression has been extensively studied, the function of ANXA1 in driving lenvatinib resistance in HCC has not yet been explored.

In this study, we demonstrate that tumor-derived ANXA1 intrinsically regulates lenvatinib resistance by promoting cancer stemness in HCC tumor cells through the activation of a STAT3/S100A6 positive feedback loop. We identify SOX2 as an upstream regulator of ANXA1 that drives this signaling pathway. Through scRNA-seq analysis and in vitro macrophage polarization experiments, we show that secreted ANXA1 induces M2 macrophage polarization via FPR2 binding and intracellular ERK and NF-κB signaling activation, facilitating T cell infiltration. In our LenR *Tp53*^KO^*/Myc*^OE^ HCC mouse model, suppression of ANXA1 using an adeno-associated virus serotype 8 (AAV8) approach in combination with lenvatinib results in maximal inhibition of HCC tumor growth and a reshaped TME that enhances T cell function. Collectively, this study highlights ANXA1 as a novel therapeutic target for combating lenvatinib resistance, regulating cancer stemness intrinsically and influencing the immune TME extrinsically.

## Results

### Identification of *ANXA1* as a crucial mediator of lenvatinib resistance in PDTX HCC via scRNA-seq analysis

**Fig. 1:**
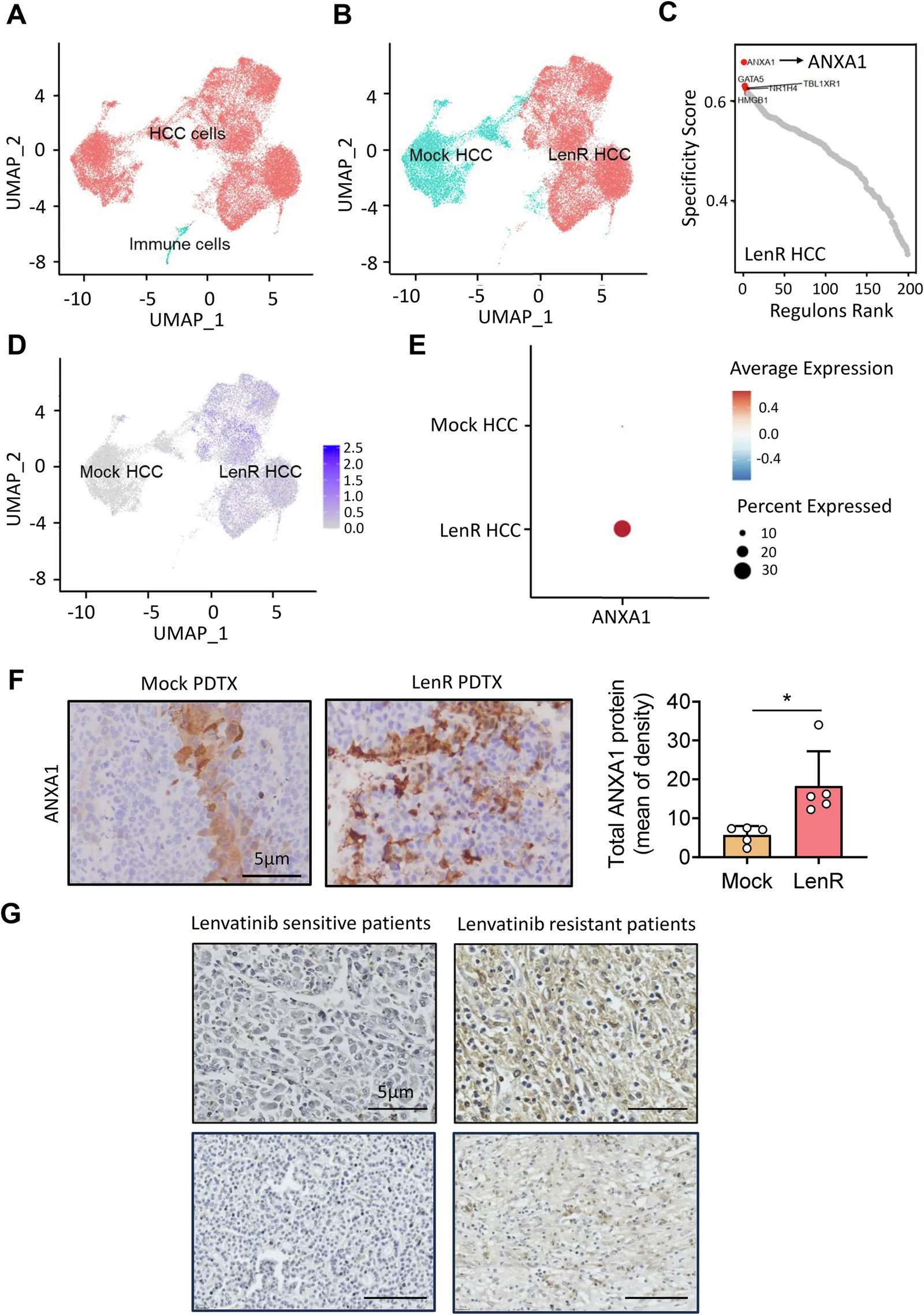
Identification of ANXA1 overexpression in LenR PDTX HCC by scRNA-seq analysis. **A** Uniform manifold approximation and projection (UMAP) visualization of defined cell types: HCC and immune cells. **B** UMAP plot showing the distribution of LenR and Mock HCC cells. **C** SCENIC analysis of LenR and Mock HCC cells. The regulon rank was set on the basis of the regulon specificity score of genes that were specifically activated in the LenR and Mock HCC cell populations. **D** Feature plot of *ANXA1* expression via UMAP plot. **E** Dot plot of average *ANXA1* expression and its percentage expression in the LenR HCC and Mock HCC cell populations. **F** IHC staining analysis of ANXA1 protein expression in LenR PDTX HCC tumors and mock control tumors. Five images were randomly taken for each sample group. ANXA1 protein expression was quantified via FIJI (ImageJ). Bar plot showing ANXA1 protein expression in sample group (*n* = 5, two-tailed *t* test). **G** IHC staining of ANXA1 expression in tumor tiusse samples from two lenvatinib-sensitive HCC and LenR HCC patients (*n* = 2 patients). Scale bar: 5 µm. Data were presented as mean ± standard deviation. *p < 0.05.

To explore the mechanisms underlying lenvatinib resistance in HCC, we previously developed LenR PDTX derived from PY003 [8], and their genetic profiles were analyzed by scRNA-seq analysis. Upon analysis, we identified HCC cells as the main population by detecting marker gene expression (Fig. 1A and Supplementary Fig. 1A). The corresponding distributions of HCC cells in the LenR and Mock groups were shown in UMAP (Fig. 1B). We performed SCENIC analysis to identify genes activated and overexpressed in the LenR group for potential therapeutic targeting, and found that ANXA1 was the most enriched gene in LenR HCC tumors (Fig. 1C). By feature plot and dot plot of *ANXA1* expression, we demonstrated that *ANXA1* expression in LenR HCC tumors was higher than that in their mock counterparts (Fig. 1D, E). Upregulation of ANXA1 expression at both the mRNA and protein levels was consistently observed in LenR PDTX tumors, as confirmed by qPCR and IHC analysis (Fig. 1F and Supplementary Fig. 1B). To verify whether ANXA1 overexpression was not restricted to only PY003 LenR tumor samples, we examined ANXA1 expression in our established LenR cell lines (PLC/PRF/5, Huh7, and MHCC-97L) through long-term administration of lenvatinib. We consistently observed the upregulation of ANXA1 in these cell lines, as demonstrated by RT-qPCR, Western blot and IHC analyses (Supplementary Fig. 1C–E). Similar results were observed in our LenR HCC mouse models (*Tp53*^KO^/*Myc*^OE^ and RIL175) [15], which were generated through the injection of plasmids containing *Tp53*^KO^ and *Myc*^OE^ via the hydrodynamic tail vein injection (HTVI) delivery method [16] and the orthotopic injection of RIL175 mouse HCC cells into the mouse liver, followed by administration of lenvatinib (Supplementary Fig. 2A, B). We also examined the change pattern of ANXA1 expression in LenR *Tp53*^KO^/*Myc*^OE^ HCC tumors. We observed a gradual increase of ANXA1 expression during the lenvatinib administration (Supplementary Fig. 2C). Finally, we analyzed ANXA1 expression in tumors from four HCC patients treated with lenvatinib, comprising two sensitive patients and two resistant patients (Supplementary Table 1). ANXA1 was upregulated in resistant patients, confirming its role in lenvatinib resistance (Fig. 1G). Collectively, ANXA1 was found to be upregulated in the LenR HCC cell line, mouse models, and patients.

### ANXA1 regulates cancer stemness-driven lenvatinib resistance

**Fig. 2:**
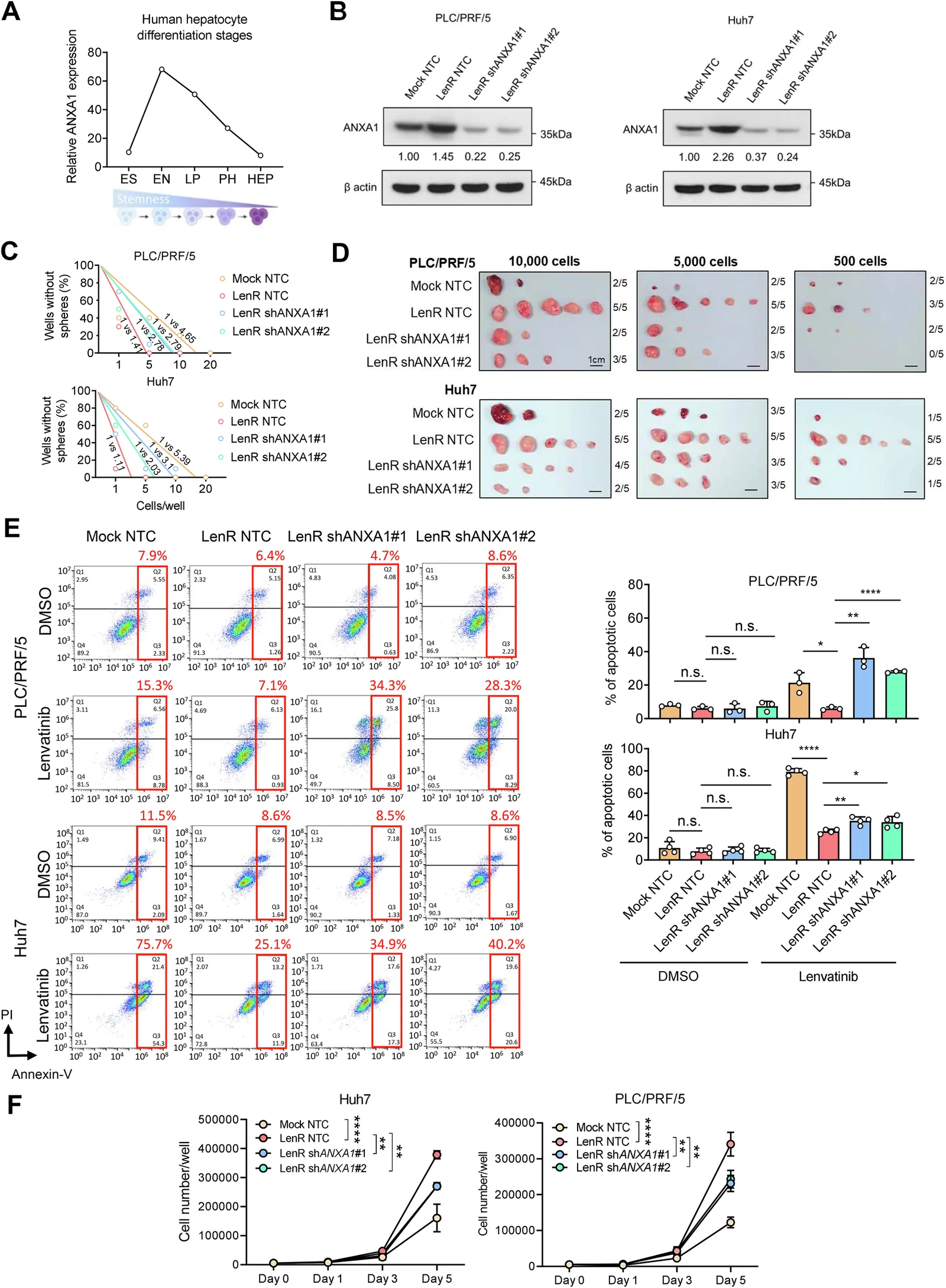
Knockdown of ANXA1 expression suppresses cancer stemness and lenvatinib resistance. **A** *ANXA1* expression at different stages of hepatocyte differentiation induced by defined culture media and growth factors in an in vitro differentiation model. ES embryonic stem cells. EN endoderm. LP luminal progenitor. PH premature hepatocytes. HEP hepatocytes (*n* = 1). **B** ANXA1 protein expression in Mock NTC, LenR NTC, LenR sh*ANXA1*#1, and LenR sh*ANXA1*#2 PLC/PRF/5 and Huh7 cells, as determined by Western blot. Human β-actin was used as a loading control (*n* = 3 independent experiments). **C** The CSC frequency of each group was calculated on the basis of formed tumor sphere number derived from Mock NTC, LenR NTC, LenR sh*ANXA1*#1 and LenR sh*ANXA1*#2 PLC/PRF/5 and Huh7 cells (*n* = 2 independent experiments, one-sided extreme limiting dilution analysis with chi-square test). **D** Representative images of tumors generated by injecting 10,000, 5000, or 500 cells derived from Mock NTC, LenR NTC, LenR sh*ANXA1*#1, LenR sh*ANXA1*#2 PLC/PRF/5 and Huh7 cells. Tumor incidence rates were indicated. **E** Representative contour diagrams illustrating the percentage of annexin V-positive Mock NTC, LenR NTC, LenR sh*ANXA1*#1, LenR sh*ANXA1*#2 PLC/PRF/5 and Huh7 cells treated with 40 µM lenvatinib. Bar plot showing the percentage of apoptotic cells after 40 µM lenvatinib treatment (*n* = 3 independent experiments, two-tailed *t* test). **F** Growth curves showing the cell proliferation (*n* = 3 independent experiments, two-tailed *t* test). Data were presented as mean ± standard deviation. *p < 0.05, **p < 0.01 & ****p < 0.0001.

We developed an in vitro differentiation model in which human embryonic stem cells were differentiated into hepatocytes with defined culture medium and growth factors [17]. We found that *ANXA1* expression was elevated in the endoderm (EN) and decreased gradually during the differentiation process into hepatocytes, indicating that ANXA1 expression is associated with stemness (Fig. 2A). Using our scRNA-seq data, we split cells into an *ANXA1* high-expressing group (*ANXA1*^High^) and an *ANXA1* low-expressing group (*ANXA1*^Low^) on the basis of the *ANXA1* mean expression value in the LenR HCC and mock HCC populations (Supplementary Fig. 3A). Our analysis revealed that, compared with the *ANXA1*^Low^ group, the *ANXA1*^High^ group presented upregulation of liver cancer stemness markers, including *CD24*, *CD47*, *BMI1*, *KLF4*, and *PROM1* (Supplementary Fig. 3B). This finding was supported by the data from The Cancer Genome Atlas Liver Hepatocellular Carcinoma (TCGA-LIHC) dataset, which showed a positive correlation between *ANXA1* expression and liver cancer stem cell marker expression (Supplementary Fig. 3C), suggesting that *ANXA1* is positively associated with cancer stemness. To examine the role of ANXA1 in cancer stemness-driven lenvatinib resistance, we suppressed ANXA1 expression in LenR PLC/PRF/5 and Huh7 cells using a lentiviral-based knockdown approach and compared the stemness properties and lenvatinib resistance of these cells with those of their corresponding nontargeted controls (NTCs) and mock controls. The successful knockdown of ANXA1 in LenR PLC/PRF/5 and Huh7 cells was confirmed by Western blot, IF staining and IHC staining analyses (Fig. 2B and Supplementary Fig. 4A–C). Suppression of differentiation markers expression, including *HNF4A* and *SERPINA1* was observed in LenR HCC cells, while knockdown of ANXA1 in these cells reversed the suppressive effects (Supplementary Fig. 4D), suggesting ANXA1 promotes a dedifferentiated state in LenR tumors. Using a limiting dilution assay, we found that, compared with mock control cells, LenR HCC cells presented a significantly greater stem cell frequency, whereas ANXA1 knockdown significantly diminished this enhancing capacity (Fig. 2C). Moreover, we demonstrated the role of ANXA1 in regulating tumorigenicity, as evidenced by the inhibition of tumorigenicity in LenR cells upon repression of this protein (Fig. 2D & Supplementary Table 2A, B). By Annexin V staining, we showed that suppression of ANXA1 significantly reduced lenvatinib resistance, as evidenced by an increased number of apoptotic cells (Fig. 2E). Furthermore, knockdown of ANXA1 significantly attenuated cell proliferation of LenR HCC cells (Fig. 2F). Consistent with these findings, the overexpression of ANXA1 in Huh7 cells showed the opposite effect (Supplementary Fig. 5A–D and Supplementary Table 2C). Next, we examined whether secretory ANXA1 also exerted similar cancer stemness-enhancing effects. To this end, we collected conditioned media (CM) from empty vector (EV) and ANXA1-overexpressing (OE) Huh7 cells and compared their effects in the aforementioned functional assays. We found that the CM from OE cells induced stem cell frequency and lenvatinib resistance compared to that from EV cells (Supplementary Fig. 6A, B), implicating the role of secretory ANXA1 in regulating these stemness properties. Together, these findings suggest that ANXA1 and its secretory form drive cancer stemness in HCC.

### Identification of S100A6 as a potential ANXA1 downstream target

**Fig. 3:**
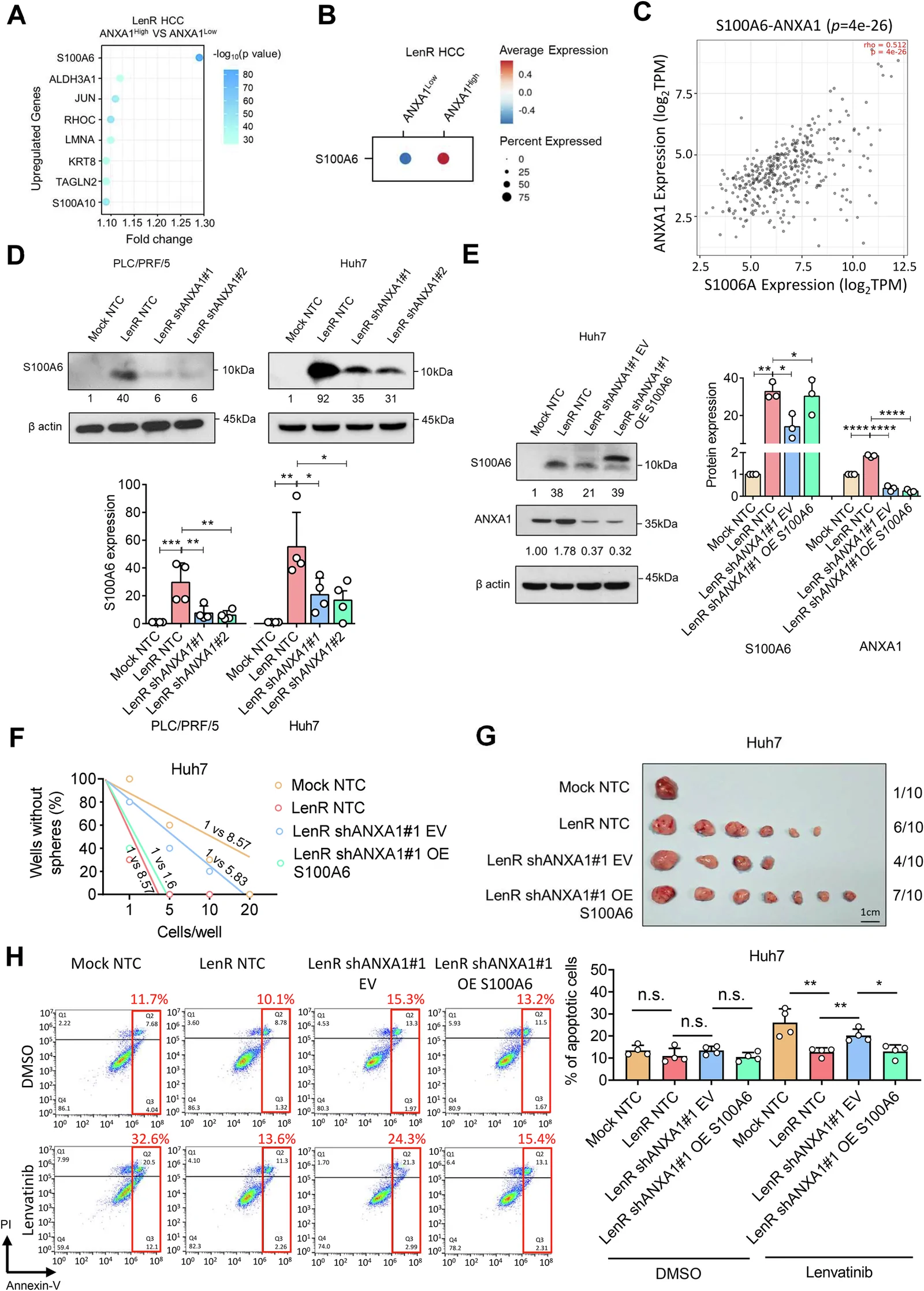
S100A6 is a downstream effector of ANXA1-driven cancer stemness and lenvatinib resistance. **A** Dot plot of the top 8 upregulated DEGs in the *ANXA1*^High^ group compared with the *ANXA1*^Low^ group in the LenR HCC population. *S100A6* was identified as the gene with the highest expression in the *ANXA1*^High^ group. **B** Dot plot of average *S100A6* expression in the *ANXA1*^High^ and *ANXA1*^Low^ groups in the LenR HCC population. **C** Positive correlation between *S100A6* and *ANXA1* expressions in the TCGA-LIHC patient cohort. TPM: transcripts per million. **D** S100A6 protein expression in Mock NTC, LenR NTC, LenR sh*ANXA1*#1, LenR sh*ANXA1*#2 PLC/PRF/5 and Huh7 cells by western blot (*n* = 4 independent experiments, one way ANOVA followed by Tukey’s multiple comparison test). **E** S100A6 and ANXA1 protein expressions in Mock NTC, LenR NTC, LenR sh*ANXA1*#1 EV, and LenR sh*ANXA1*#1 OE S100A6 Huh7 cells by western blot (*n* = 3 independent experiments, one way ANOVA followed by Tukey’s multiple comparison test). **F** Limiting dilution assay for calculating the CSC frequency of Mock NTC, LenR NTC, LenR shANXA1#1 EV, and LenR sh*ANXA1*#1 OE S100A6 Huh7 cells (*n* = 2 independent experiments, one-sided extreme limiting dilution analysis with chi-square test). **G** Representative images of tumors generated by injecting 200,000 cells derived from Mock NTC, LenR NTC, LenR sh*ANXA1*#1 EV, and LenR sh*ANXA1*#1 OE S100A6 Huh7 cells. Tumor incidence rates were indicated. **H** Representative contour diagrams illustrating the percentage of annexin V-positive Mock NTC, LenR NTC, LenR sh*ANXA1*#1 EV, and LenR sh*ANXA1*#1 OE S100A6 Huh7 cells under 40 µM lenvatinib treatment. Bar plot showing the percentage of apoptotic cells after 40 µM lenvatinib (*n* = 4 independent experiments, two-tailed *t* test). Data were presented as mean ± standard deviation. *p < 0.05, **p < 0.01, ***p < 0.001, ****p < 0.0001.

We identified downstream targets of *ANXA1* by comparing differentially expressed genes (DEGs) in the *ANXA1*^High^ and *ANXA1*^Low^ groups of LenR PDTX HCC cells. *S100A6* was most enriched in the *ANXA1*^High^ group (Fig. 3A, B), suggesting that *ANXA1* may regulate its expression. In the TCGA-LIHC dataset, *ANXA1* and *S100A6* were found to be frequently overexpressed in HCC compared with the normal liver, and overexpression of *ANXA1* and *S100A6* were correlated with advanced tumor stage (T4 versus T1/2/3) (Supplementary Fig. 7A). Furthermore, a positive correlation between *ANXA1* and *S100A6* expression was revealed (Fig. 3C). Additionally, S100A6 expression was upregulated in three LenR HCC cell lines: PLC/PRF/5, Huh7, and MHCC-97L (Supplementary Fig. 7B–D). Knockdown of ANXA1 expression reduced S100A6 level, as shown by Western blot (Fig. 3D), further indicating that ANXA1 may regulate S100A6 expression. By scRNA-seq analysis, we divided the LenR PDTX HCC cell population into *S100A6*^High^ and *S100A6*^Low^ groups (Supplementary Fig. 8A). The *S100A6*^High^ group exhibited upregulation of various stemness markers, as shown in the dot plot (Supplementary Fig. 8B). Such correlation was also observed in TCGA-LIHC cohort (Supplementary Fig. 8C). Additionally, CytoTRACE analysis of our scRNA-seq data revealed that *S100A6* expression was consistently higher in high-stemness clusters than in low-stemness clusters (Supplementary Fig. 8D). To further investigate the role of S100A6 as a potential ANXA1 downstream target in regulating cancer stemness and lenvatinib resistance, we successfully established LenR sh*ANXA1*#1 cells with S100A6 overexpression, as shown by Western blot (Fig. 3E). In vitro limiting dilution and in vivo tumorigenicity assays demonstrated that S100A6 overexpression offset the suppressive effects on self-renewal and tumorigenicity in LenR sh*ANXA1*#1 cells (Fig. 3F, G). Additionally, we found that S100A6 is crucial for ANXA1-driven lenvatinib resistance (Fig. 3H). As S100A6 was previously reported to regulate β-catenin activity [18,19], we examined this pathway and found that ANXA1 knockdown decreased β-catenin, whereas S100A6 overexpression markedly increased it, suggesting the ANXA1/S100A6 axis may promote HCC stemness via β-catenin (Supplementary Fig. 8E). Overall, S100A6 overexpression mitigated the effects of ANXA1 knockdown on cancer stemness and lenvatinib resistance.

### ANXA1 regulates S100A6 expression via activation of STAT3 signaling pathway

**Fig. 4:**
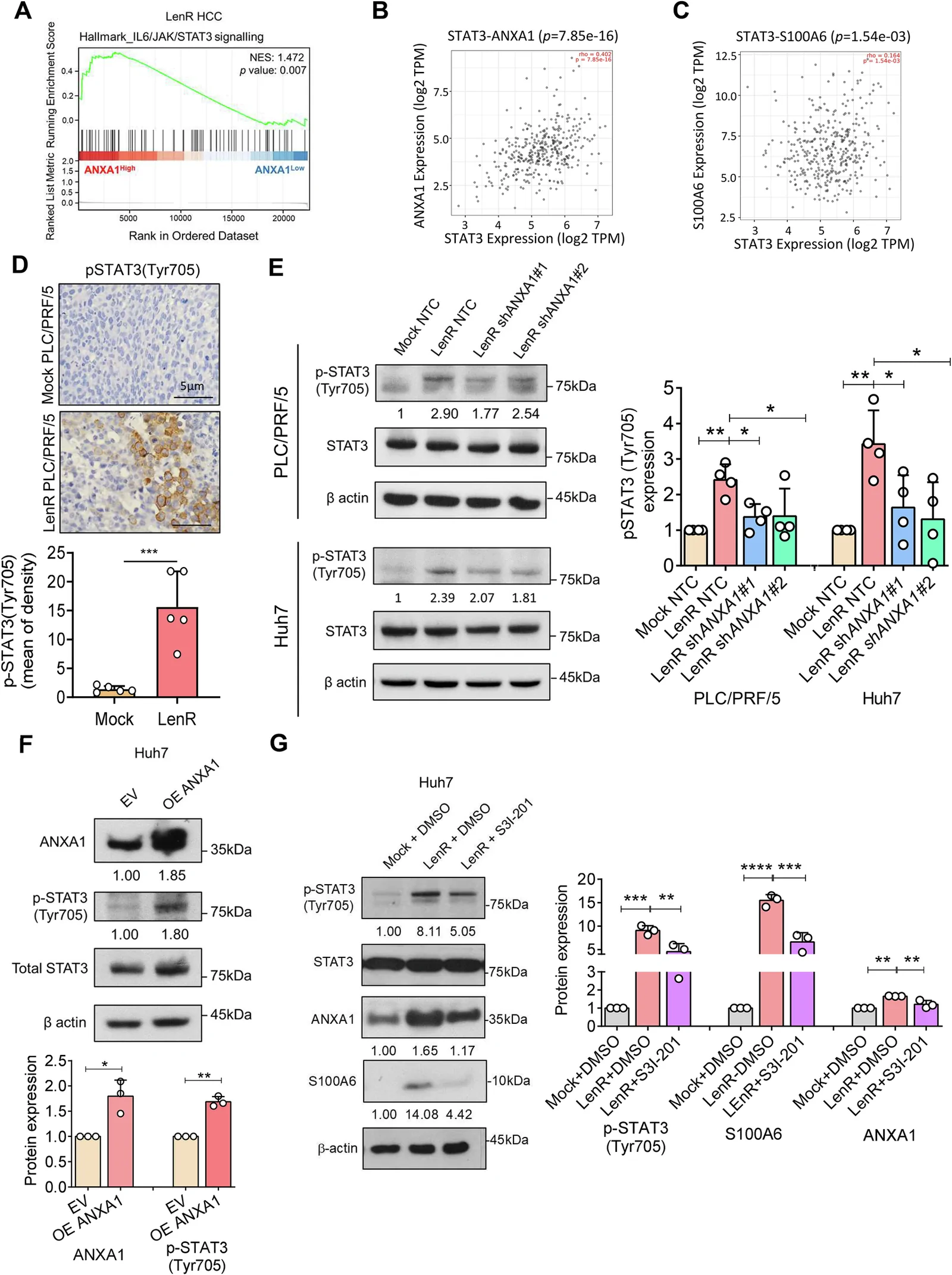
ANXA1 regulates STAT3/S100A6 signaling cascade. **A** Identification of upregulated IL-6/JAK/STAT3 signaling pathway via GSEA of DEGs in the *ANXA1*^High^ group compared with the *ANXA1*^Low^ group in the LenR HCC cell population. **B** Correlation analysis between *STAT3* and *ANXA1* in the TCGA-LIHC patient cohort. **C** Correlation analysis between *STAT3* and *S100A6* expression in the TCGA-LIHC patient cohort. **D** IHC staining analysis of p-STAT3 protein expression in tumor tissue samples derived from LenR and Mock PLC/PRF/5 cells. Five images were randomly taken for each sample group. p-STAT3 protein expression was quantified by FIJI (ImageJ). Bar plot showing p-STAT3 protein expression (*n* = 5, two-tailed *t* test). **E** Western blot showing p-STAT3 (Tyr705) and total STAT3 protein expression in Mock NTC, LenR NTC, LenR sh*ANXA1*#1, LenR sh*ANXA1*#2 (*n* = 4 independent experiments, one way ANOVA followed by Tukey’s multiple comparison test). **F** Western blot analysis of ANXA1, p-STAT3 (Tyr705), and total STAT3 protein expression in Huh7 EV and OE ANXA1 cells (*n* = 3 independent experiments, one way ANOVA followed by Tukey’s multiple comparison test). **G** Western blot showing p-STAT3 (Tyr705), total STAT3, ANXA1 and S100A6 protein expression in LenR and Mock Huh7 cells treated with 100 µM S3I-201 or DMSO (*n* = 3 independent experiments, one-way ANOVA followed by Tukey’s multiple comparison test). Data were presented as mean ± standard deviation. *p < 0.05. **p < 0.01, ***p < 0.001, ****p < 0.0001.

By performing gene set enrichment analysis (GSEA) of DEGs when comparing the *ANXA1*^High^ group to the *ANXA1*^Low^ group using our scRNA-seq data, we identified the upregulation of IL6/JAK/STAT3 signaling pathway (Fig. 4A). Consistently, *ANXA1* was positively correlated with *IL6;* and alternation of ANXA1 changed pJAK2 (Tyr1007/1008) expression (Supplementary Fig. 9A, B). In addition, we also observed a positive correlation between *STAT3* and *ANXA1*/*S100A6* expression (Fig. 4B, C), suggesting that STAT3 might be involved in the ANXA1/S100A6 signaling cascade. By IHC staining analysis, we detected an increased level of p-STAT3(Tyr705) in tumors derived from LenR PLC/PRF/5 cells (Fig. 4D). To further investigate whether ANXA1 can regulate the activation of STAT3 signaling, we performed western blot to examine the expression of p-STAT3(Tyr705) and total STAT3. Knockdown of ANXA1 expression in LenR HCC cells suppressed p-STAT3(Tyr705) expression (Fig. 4E), whereas overexpression of ANXA1 in Huh7 cells increased p-STAT3(Tyr705) expression (Fig. 4F). Furthermore, we utilized the p-STAT3 inhibitor S3I-201 to suppress p-STAT3 expression. We found a decrease in S100A6 and ANXA1 expression with the suppression of p-STAT3 by using S3I-201 in LenR and ANXA1-overexpressing cells, indicating that STAT3 signaling might be able to regulate S100A6 expression (Fig. 4G and Supplementary Fig. 10A). This data, together with the presence of putative STAT3 binding sites in the S100A6 promoter (Supplementary Supplementary Fig. 10B–D), suggests that ANXA1 may regulate S100A6 expression through direct transcriptional activation mediated by STAT3. Notably, S3I-201 effectively reversed the resistant phenotype in both LenR Huh7 cells and ANXA1-overexpressing cells, thereby restoring their sensitivity to lenvatinib treatment. (Supplementary Fig. 10E). This data reinforces the role of the ANXA1/STAT3 axis in regulating S100A6-mediated resistance to lenvatinib.

### Identification of SOX2 as a novel ANXA1 upstream regulator

**Fig. 5:**
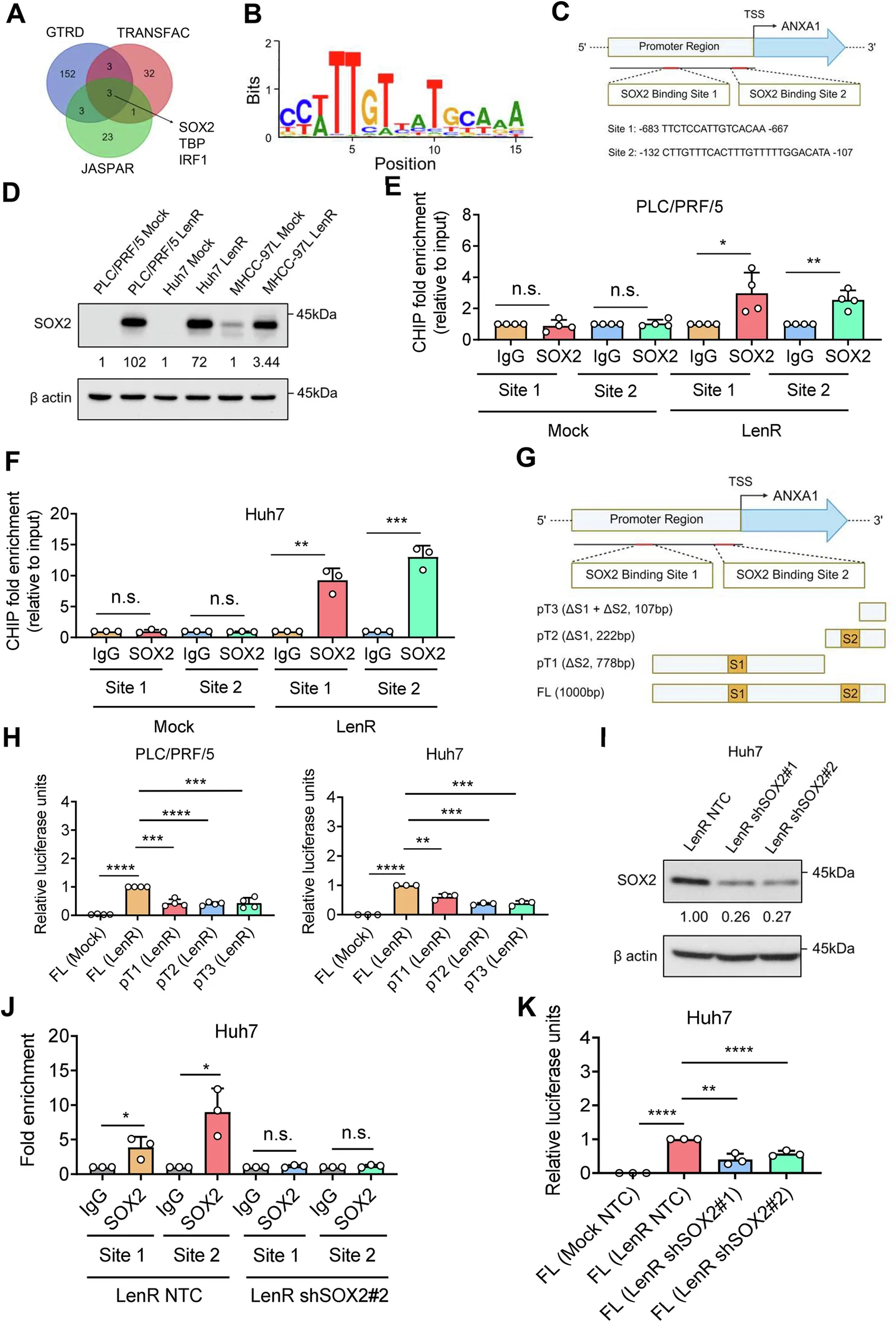
Identification of SOX2 as an ANXA1 upstream regulator. **A** Venn diagram illustrating SOX2 as the common TF among three TF-binding site databases: GTRD, TRANSFAC and JASPAR. **B** Consensus binding motif of SOX2. **C** Schematic diagram of two predicted SOX2 binding sites (Site 1 at −683 to −667 bp and Site 2 at −132 to −107 bp) in the ANXA1 promoter region, as predicted by GTRD, TRANSFAC and JASPAR. TSS: transcription start site. **D** SOX2 protein expression in LenR and their mock controls. **E**, **F** ChIP-qPCR confirmed that SOX2 binds to two predicted sites (Site 1 and Site 2) in the ANXA1 promoter region in LenR PLC/PRF/5 and Huh7 cells with high SOX2 expression using SOX2 and IgG antibodies. Mock PLC/PRF/5 and Huh7 cells harboring low SOX2 expression were used for comparison (*n* = 4 independent experiments (PLC/PRF/5), *n* = 3 independent experiments (Huh7), two-tailed *t* test). **G** Schematic diagram showing the reporter constructs created by cloning FL or truncated ANXA1 promoter fragments (pT1, pT2, and pT3) into a pGL3-Basic vector that encodes firefly luciferase. **H** Luciferase reporter assay showing the relative luciferase activity (firefly/renilla) in LenR and Mock PLC/PRF/5 and Huh7 cells with FL or truncated ANXA1 promoters (pT1, pT2, and pT3). The pRL-CMV renilla luciferase plasmid was co-transfected for normalization (*n* = 4 independent experiments (PLC/PRF/5), *n* = 3 independent experiments (Huh7), two-tailed *t* test). **I** SOX2 protein expression in LenR NTC, LenR sh*SOX2*#1, and LenR sh*SOX2*#2 Huh7 cells. **J** ChIP-qPCR assay showing that knockdown of SOX2 expression suppressed SOX2 binding to predicted sites (Site 1 and Site 2) in the ANXA1 promoter in LenR Huh7 cells (*n* = 3 independent experiments, two-tailed *t* test). **K** Luciferase reporter assay indicating the relative luciferase activity (firefly/renilla) in Mock NTC, LenR NTC, LenR sh*SOX2*#1, and LenR sh*SOX2*#2 Huh7 cells with FL of the ANXA1 promoter. The pRL-CMV renilla luciferase plasmid was co-transfected for normalization (*n* = 3 independent experiments, two-tailed *t* test). Data was presented as mean ± standard deviation. *p < 0.05, **p < 0.01, ***p < 0.001 and ****p < 0.0001.

To identify the possible upstream regulatory elements that lead to ANXA1 overexpression in HCC, transcription factors (TFs) that bind to the ANXA1 promoter were predicted using three online available TF-binding site databases: Gene Transcription Regulation Database (GTRD), TRANScription FACtor (TRANSFAC), and JASPAR. SOX2, one of the three transcription factors, was commonly found among the three databases and was identified to harbor two high-scoring predicted binding sites, TTCTCCATTGTCACAA at −683 to −667 and CTTGTTTCACTTTGTTTTTGGACATA at −132 to −107 (Fig. 5A-C). Similar to ANXA1, the overexpression of SOX2 in three LenR HCC cell lines was apparent, as demonstrated by RT-qPCR, Western blot and IHC staining analyses (Fig. 5D and Supplementary Fig. 11A–C). By correlation analysis, we identified a positive correlation between ANXA1 and SOX2 expression in TCGA-LIHC dataset for HCC patients (Supplementary Fig. 11D). Using a chromatin immunoprecipitation-quantitative PCR (ChIP-qPCR) assay, we demonstrated that SOX2 binds to two predicted sites on the ANXA1 promoter, as evidenced by a significant enrichment in SOX2 binding compared with that of ChIP with nonspecific IgG (Fig. 5E, F). To further confirm the significance of these two binding sites, we created three truncated mutants (pT1, pT2, and pT3) through serial deletion of ANXA1 promoter fragments that lacked one or both SOX2 binding sites (Fig. 5G). A notable decrease in luciferase activity was detected when one or both predicted binding sites were truncated, indicating that these two SOX2 binding sites are essential for regulating ANXA1 transcription (Fig. 5H). Next, we constructed SOX2 stable knockdown clones in LenR Huh7 cells, as shown by Western blot (Fig. 5I and Supplementary Fig. 11E). The use of a lentiviral-based transfection strategy to stably knockdown SOX2 consistently led to a significant decrease in SOX2 binding to the ANXA1 promoter, as measured by a ChIP-qPCR assay (Fig. 5J), and a noticeable reduction in luciferase activity (Fig. 5K). Supporting evidence shows that STAT3 can regulate SOX2 expression via direct binding to the SOX2 promoter region [20]. Interestingly, we also found a potential binding site for STAT3 in the SOX2 promoter region using the GTRD database (Supplementary Fig. 12A, B). We also identified a positive correlation between SOX2 and STAT3 expression in the TCGA-LIHC patient cohort (Supplementary Fig. 12C). We further validated whether the phosphorylated form of STAT3 in the nucleus could regulate the expression of SOX2 by using the p-STAT3 inhibitor S3I-201. Upon the suppression of p-STAT3, decreased expression of SOX2 was observed by western blot (Supplementary Fig. 12D), indicating that SOX2 expression could be regulated by STAT3. We also noticed a decrease in SOX2 expression with ANXA1 knockdown (Supplementary Fig. 12E). These findings implicate that SOX2/ANXA1/STAT3/S100A6 potentially forms a positive feedback loop in regulating cancer stemness and lenvatinib resistance.

### Secretory ANXA1 from LenR HCC cells drives M2 macrophage polarization in vitro

**Fig. 6:**
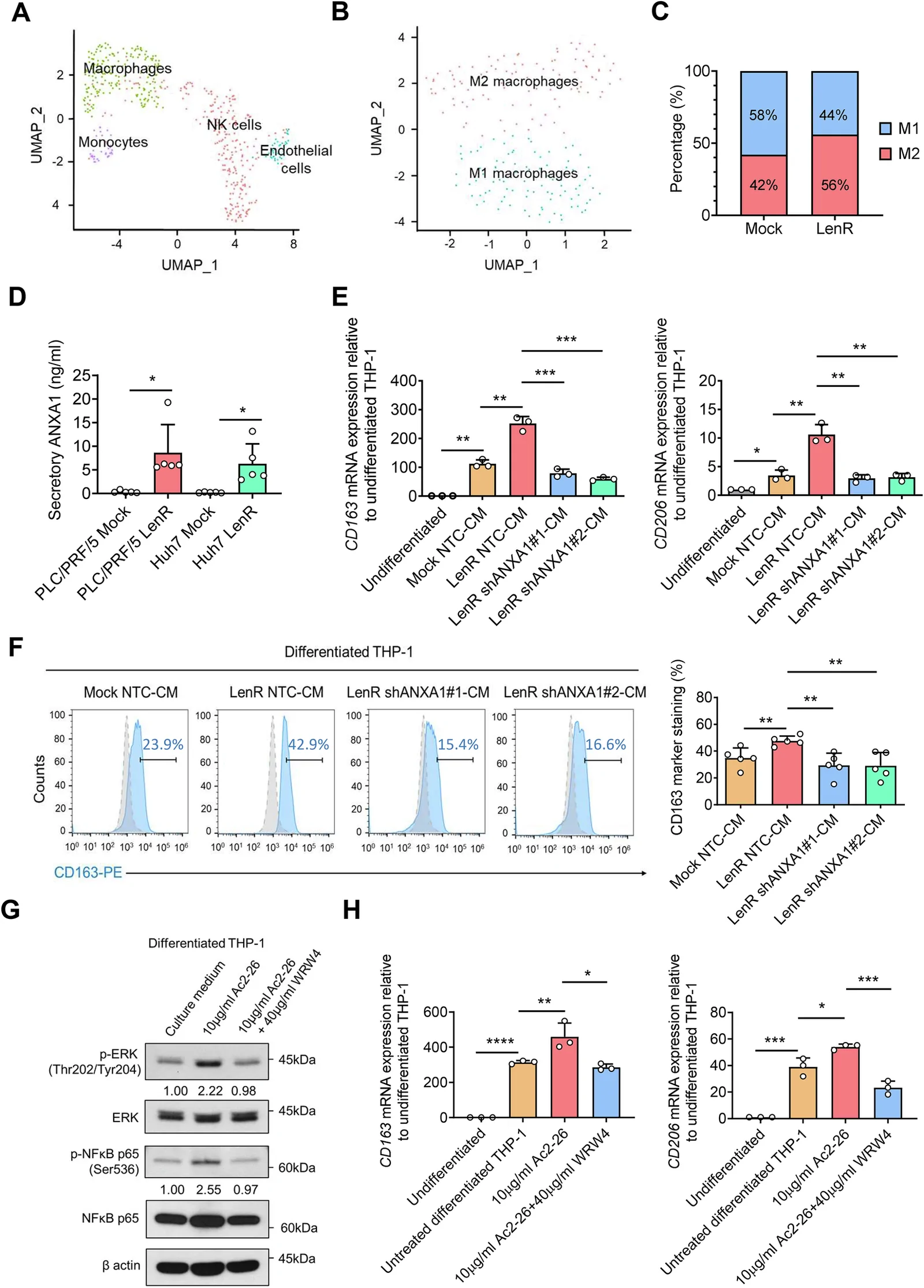
LenR HCC cell-derived ANXA1 drives M2 macrophage polarization in vitro. **A** UMAP plot of defined cell types: monocytes, macrophages, NK cells and endothelial cells in the mouse cell population. **B** UMAP plot of M1 and M2 macrophages in the mouse macrophage population in Mock and LenR PY003 HCC tumors. **C** Bar plot showing the percentage of M1 and M2 macrophages in the mouse LenR and Mock cell populations. **D** Secretory ANXA1 concentrations in LenR PLC/PRF/5 and Huh7 and their mock counterparts detected via ELISA (*n* = 5 independent experiments, two-tailed *t* test). **E** *CD163* and *CD206* mRNA expression fold change in differentiated THP-1 cells treated with conditioned media (CM) from Mock NTC, LenR NTC, LenR sh*ANXA1*#1, and LenR sh*ANXA1*#2 PLC/PRF/5 cells (*n* = 3 independent experiments, two-tailed *t* test). **F** Percentage of CD163^+^ cells among differentiated THP-1 cells after incubation with CM derived from Mock NTC, LenR NTC, LenR sh*ANXA1*#1, and LenR sh*ANXA1*#2 PLC/PRF/5 cells (*n* = 5 independent experiments, two-tailed *t* test). **G** Western blot analysis of p-ERK (T202/Y204), ERK, p-NF-κB p65 (Ser536), and NF-κB p65 protein expression in differentiated THP-1 cells treated with 10 µg/ml Ac2-26 or 10 µg/ml Ac2-26 + 40 µg/ml WRW4 in THP-1 culture medium. **H** *CD163* and *CD206* mRNA expression fold change in differentiated THP-1 cells treated with either 10 µg/ml Ac2-26 or 10 µg/ml Ac2-26 + 40 µg/ml WRW4 (*n* = 3 independent experiments, two-tailed *t* test). Data were presented as mean ± standard deviation. *p < 0.05, **p < 0.01, ***p < 0.001, ****p < 0.0001.

To investigate how LenR HCC tumors reshape the TME, we analyzed mouse cells surrounding human PY003 HCC tumors via the same set of scRNA-seq data. We identified monocytes, macrophages, NK cells and endothelial cells on the basis of marker gene expression (Fig. 6A and Supplementary Fig. 13A). As macrophages play a key role in modulating the TME, we further examined the macrophage population using our scRNA-seq data. We identified M1 and M2 macrophages based on marker gene expression (Fig. 6B and Supplementary Fig. 13B). We found a higher M2/M1 macrophage ratio in the mouse LenR cell population than in the mock counterpart (Fig. 6C and Supplementary Fig. 13C). To validate increased M2 macrophage infiltration in LenR HCC tumors, we conducted IHC staining for the M2 marker CD163 in three LenR HCC models (LenR PDTX, LenR *Tp53*^KO^/*Myc*^OE^, and LenR RIL175) and their mock counterparts. The results showed a significantly increased M2 macrophage infiltration in LenR HCC tumors (Supplementary Fig. 14A–C). An analysis of the TCGA-LIHC patient cohort revealed that *ANXA1* expression was positively correlated with M2 macrophage infiltration and the expression of M2 macrophage markers *CD163*, *MRC1(CD206)*, and *CD36* (Supplementary Fig. 14D, E). To verify the extrinsic role of ANXA1 in modulating the composition of macrophage population, we first measured the concentration of secretory ANXA1 in the CM of ANXA1-altered HCC cells. We consistently observed an increase in secretory ANXA1 in the CM of LenR PLC/PRF/5 and Huh7 HCC cells (Fig. 6D). Conversely, the secretion of ANXA1 decreased upon the suppression of ANXA1 expression (Supplementary Fig. 15A). To further investigate the role of secretory ANXA1 from LenR HCC cells in M2 macrophage polarization, we treated M0 macrophages, which were previously differentiated from THP-1 monocytes, with CM derived from ANXA1-altered HCC cells. RT-qPCR revealed that ANXA1 knockdown significantly suppressed the mRNA expression of *CD163* and *CD206* (Fig. 6E), indicating that ANXA1 promotes M2 macrophage polarization. In addition, we used flow cytometry to detect the expression of CD163 and the M1 macrophage surface marker CD86. Our results showed that the percentage of M2 macrophages significantly increased when differentiated THP-1 cells were incubated with CM derived from LenR HCC cells compared with mock cells, whereas knockdown of ANXA1 in LenR HCC cells inhibited this effect (Fig. 6F). In contrast, the percentage of M1 macrophages was significantly lower in CM from LenR HCC cells than in that from mock control and LenR HCC cells with ANXA1 knockdown (Supplementary Fig. 15B). We further investigated whether ANXA1 can regulate macrophage polarization via the N-terminal domain of ANXA1 interacting with FPR2 on the macrophage surface by using a mimetic peptide of the ANXA1 N-terminal domain named Ac2-26 and the FPR2 antagonist WRW4. Upon Ac2-26 treatment, ERK and NF-κB signaling were activated in THP-1-derived macrophages, while blockage of FPR2 via WRW4 abolished this effect (Fig. 6G and Supplementary Fig. 15C). We further confirmed that M2 macrophage polarization occurred upon Ac2-26 treatment and was abolished by the addition of WRW4 (Fig. 6H), indicating that N-terminal ANXA1 and FPR2 play crucial roles in modulating macrophage polarization (Supplementary Fig. 15D). These findings suggest that a crucial role of secretory ANXA1 from LenR HCC cells in driving the M2 macrophages polarization.

### In vivo knockdown of *Anxa1* via the AAV8 approach sensitizes HCC cells to lenvatinib treatment in a LenR *Tp53*^KO^/*Myc*^OE^ HCC mouse model

**Fig. 7:**
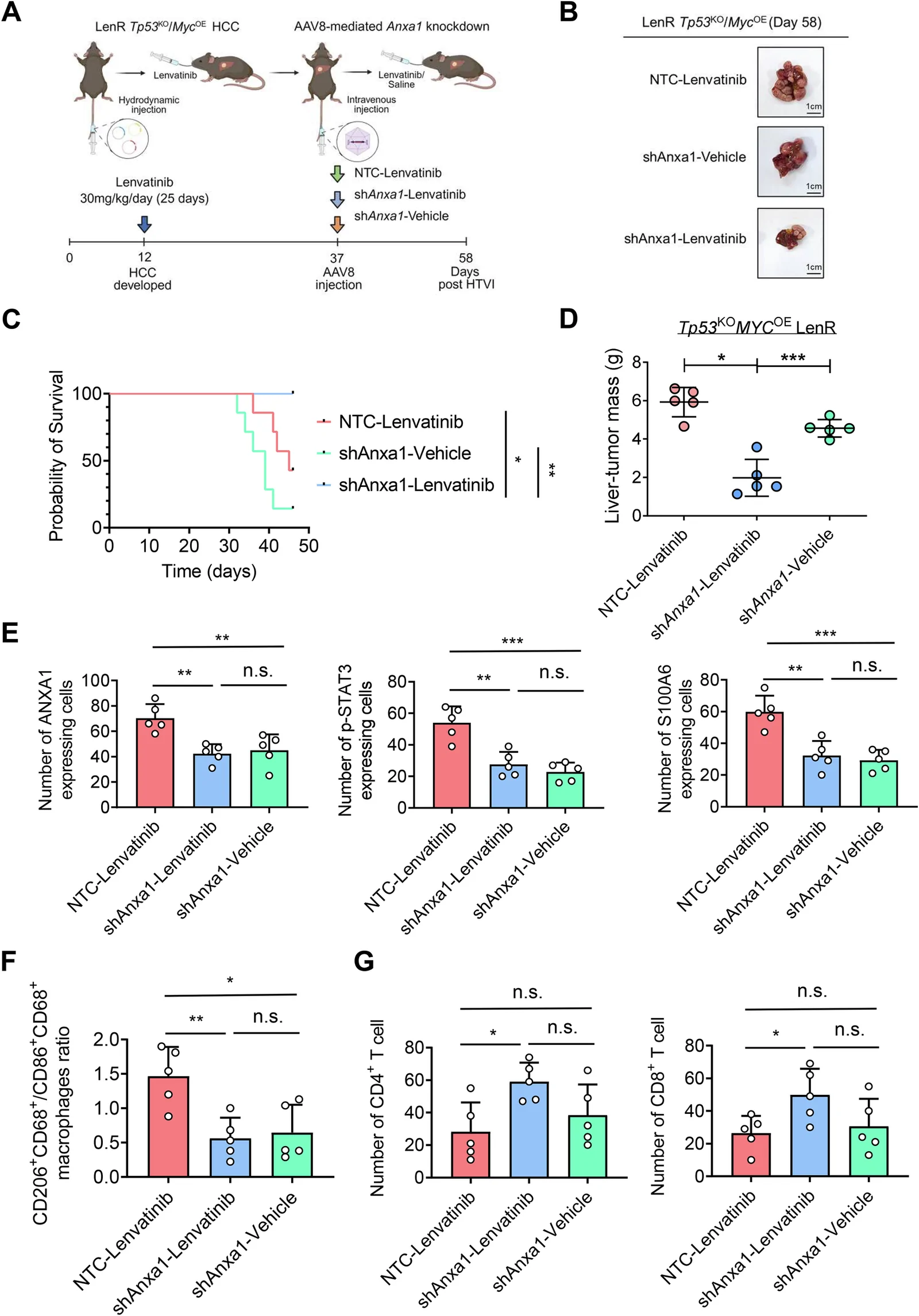
The effect of *Anxa1* knockdown in suppressing tumor growth and prolonging survival. **A** Schematic diagram of the treatment regimens of NTC-Lenvatinib (NTC shRNA + 30 mg/kg/day lenvatinib), sh*Anxa1*-Vehicle (AAV8-mediated shAnxa1 + saline), and sh*Anxa1*-Lenvatinib (AAV8-mediated sh*Anxa1* + 30 mg/kg/day lenvatinib). **B** Representative images of HCC tumors derived from these three groups at the endpoint. Scale bar: 1 cm. **C** Survival curves of the mice in these three groups (*n* = 7 mice from the NTC-Lenvatinib group, *n* = 7 mice from the sh*Anxa1*-Vehicle group, and *n* = 6 mice from the sh*Anxa1*-Lenvatinib group). **D** Dot plot showing the liver-tumor mass of the mice at the endpoint (*n* = 5 mice per group, one way ANOVA followed by Tukey’s multiple comparison test). **E** Multiplex staining analysis of ANXA1, pSTAT3 Tyr(705) andS100A6 expression in HCC mouse tissue samples. Five images were randomly taken for each treatment group. The number of ANXA1^+^, pSTAT3 Tyr(705)^+^ and S100A6^+^ cells was quantified via FIJI (ImageJ). Bar plot showing the number of ANXA1^+^ and S100A6^+^ cells (*n* = 5, two-tailed *t* test). **F**, **G** Multiplex staining analysis of CD206^+^CD68^+^ M2 macrophages, CD86^+^CD68^+^ M1 macrophages, CD4^+^ T cells, and CD8^+^ T cells in HCC mouse tissue samples. Five images were randomly taken for each treatment group. The numbers of CD206^+^CD68^+^, CD86^+^CD68^+^, CD4^+^, and CD8^+^ cells were quantified via FIJI (ImageJ). Bar plot showing the ratio of CD206^+^CD68^+^ M2 macrophages/CD86^+^CD68^+^ M1 macrophages and the number of CD4^+^ and CD8^+^ T cells (*n* = 5, two-tailed *t* test). Data were presented as mean ± standard deviation. *p < 0.05, **p < 0.01 and ***p < 0.001.

Next, we further examined the therapeutic efficacy of Anxa1 knockdown in sensitizing LenR HCC cells in an immunocompetent mouse model. HCC was induced in wild-type C57BL/6 J mice via *Myc* overexpression and *p53* knockout using HTVI. At 12 days postinjection, the mice were administered 30 mg/kg/day lenvatinib for 25 days to mimic the clinical situation in which lenvatinib-nonresponsive patients with HCC progress after lenvatinib treatment (Fig. 7A). At this point, the mice were divided into 3 groups, as shown in Fig. 7A. The efficacy of the combined treatment was evaluated by the tumoral liver mass and survival rate compared with those of the single-drug treatment groups. Treatment with *shAnxa1* and lenvatinib led to maximal suppression of tumor growth and prolonged survival (Fig. 7B–D), indicating that suppressing ANXA1 expression synergizes with lenvatinib treatment and is effective against liver tumors in vivo. Accompanying these changes, we confirmed the Anxa1 knockdown in vivo and observed a concomitant decrease in p-STAT3 and S100A6 levels by multiplex staining analysis (Fig. 7E and Supplementary Fig. 16). Additionally, we detected a reduction in the M2/M1 macrophage ratio following Anxa1 suppression, as evidenced by the ratio of CD206^+^CD68^+^/CD86^+^CD68^+^ macrophage staining (Fig. 7F and Supplementary Fig. 17A). Echoing these findings, we detected an increased CD4^+^ and CD8^+^ T cell infiltration in Anxa1-suppressed tumors, which indicates that this TME favors T cell function (Fig. 7G and Supplementary Fig. 17B). These findings demonstrate that silencing ANXA1 sensitizes HCC to lenvatinib and remodels the immune landscape in a lenvatinib-resistant model.

## Discussion

By using scRNA-seq analysis of the LenR PDTX HCC model, we identified ANXA1 as a new resistance mechanism for lenvatinib in HCC. This finding was further validated by the consistent overexpression of ANXA1 observed in LenR HCC cell lines and various mouse models. Notably, despite the scarcity of clinical specimens, we observed elevated ANXA1 levels in two samples from LenR HCC patients compared to those from sensitive patients. To further establish ANXA1 as a predictive biomarker for lenvatinib sensitivity, additional clinical cohorts are needed. To date, few studies have focused on the involvement of tumor-derived ANXA1 in HCC development. One research study shows a significantly increased expression of ANXA1 in HCC tissues compared with adjacent noncancer tissues and reveals the function of ANXA1 in promoting cell migration and invasion via a knockdown approach [21]. High ANXA1 protein expression is also found to be associated with poorer overall survival among 160 HCC patients.

Accumulating evidence has shown that ANXA1 is associated with multidrug resistance. For instance, in triple-negative breast cancer (TNBC), compared with MCF7 cells lacking ANXA1, MDA-MB-231 cells highly expressing ANXA1 exhibit significantly increased (3- to 20-fold) resistance to etoposide, adriamycin, and melphalan [22]. In addition, ANXA1 acquires and maintains cancer stem cell (CSC) features in prostate cancer cells that are resistant to zoledronic acid (ZA) [23]. Similar to our findings, ANXA1 overexpression was observed in ZA-resistant cells, and ANXA1 knockdown abolished their resistance to ZA and suppressed their EMT-mediated invasion ability. Although these findings suggest that ANXA1 plays a role in the regulation of CSCs and drug resistance, the role of ANXA1 in these functional properties in HCC is largely unknown. Through knockdown and overexpression approaches, we demonstrated that ANXA1 is critically involved in the regulation of liver stemness and lenvatinib resistance. We also identified a novel ANXA1 upstream regulator, SOX2, via promoter activation and a new ANXA1/S100A6/STAT3 signaling cascade. However, this study did not investigate the mechanism by which SOX2 is upregulated in response to lenvatinib. Because lenvatinib induces HIF-1α-mediated hypoxia [7,24] and SOX2 has been reported as HIF-1α target [25], we will further examine whether HIF-1α regulates SOX2 induction following lenvatinib treatment. S100A6, also known as calcyclin, is a Ca^2+^-binding protein from the S100 family harboring two EF-hand calcium-binding motifs [26]. S100A6 has been reported to be associated with cancer development and CSCs [18,27]. High expression of S100A6 was identified in a subpopulation of CD24^+^/CD44^+^ CSC-enriched HCC cells via scRNA-seq analysis [28]. Based on our data, S100A6 may regulate cancer stemness through regulation of Wnt/β-catenin, but the exact mechanism is yet to be elucidated. With respect to STAT3, echoed with our findings, one study shows that ANXA1 regulates IL-6/JAK2/STAT3 signaling pathway to promote tumor proliferation and migration in papillary thyroid carcinoma [29].

In this study, we demonstrated an important intrinsic role for tumor regulatory ANXA1 in immune compromised mouse models. However, these mice still contain macrophages, allowing us to analyze macrophage populations using the same scRNA-seq dataset. Upon analysis. we revealed the potential extrinsic role of secretory ANXA1 in M2 macrophage polarization via FPR2 binding and ERK and NF-κB signaling activation. Other studies have reported findings similar to ours. Macrophages from ANXA1^−/−^ mice show reduced sensitivity to IL4-induced M2 polarization [30]. Additionally, when macrophages are treated with exogenous ANXA1, they release IL-10 and suppress inducible nitric oxide synthase (iNOS) expression, polarizing to the M2 phenotype [31]. Furthermore, when macrophages differentiated from THP-1 cells are incubated with extracellular vesicles (EVs) isolated from ANXA1 knockout pancreatic cancer cells, the macrophages fail to acquire a protumor M2 phenotype [32]. To further examine the effect of ANXA1 in regulation of macrophages mimicking heterogeneity of HCC tumors, we evaluated the potential therapeutic efficacy of Anxa1 knockdown in combination with lenvatinib in an immune-competent LenR *Tp53*^KO^/*Myc*^OE^ HCC mouse model. Suppression of ANXA1 expression combined with lenvatinib treatment provided a synergistic effect in tumor growth volume reduction and survival. Recently, Medannex has developed humanized IgG1 monoclonal antibodies that can specifically target ANXA1 by binding to ANXA1 to disrupt its interaction with FPR1/2. MDX-124 markedly decreases cell proliferation in a dose-dependent manner across various human cancer cell lines expressing ANXA1 [33]. This anti-proliferative effect is triggered by halting cell cycle progression, causing cancer cells to accumulate in the G1 phase. Additionally, MDX-124 significantly suppresses tumor growth in both 4T1-luc TNBC and Pan02 pancreatic cancer. Currently, MDX-124 is undergoing a phase 1b clinical trial in the United Kingdom [34]. In the future, if MDX-124 is available on the market, it would be valuable to test whether combining MDX-124 with lenvatinib could provide a synergistic effect in inhibiting HCC tumor growth and relieving lenvatinib resistance. If the results are favorable, our study might provide translational impact in developing a novel combination therapy for HCC.

In conclusion, we demonstrate that tumor-derived ANXA1 regulates lenvatinib resistance by modulating the SOX2/STAT3/S100A6 positive feedback loop and reshaping the immune TME in HCC (Supplementary Fig. 18). Targeting ANXA1 may offer a potential combinatorial approach alongside lenvatinib or immune checkpoint therapies for HCC treatment.

## Materials and methods

### Study approval

Clinical samples from both lenvatinib-sensitive and LenR HCC tumors were collected from Dr. Qian Qian Yu at Tongji Hospital, Tongji Medical College, Huazhong University of Science and Technology in Wuhan, China, following approval from the University’s Institutional Review Board for ethical review (2019s1096). Patient informed consent was obtained. The license to conduct animal experiments was granted by the Department of Health in Hong Kong SAR, and animal work was approved by the Animal Subjects Ethics Sub-Committee at the Hong Kong Polytechnic University.

### Statistical analysis

The statistical significance of results from western blot analyses, annexin V apoptosis assay, flow cytometry, qRT-PCR, luciferase reporter assay, ELISA, immunohistochemical and immunofluorescence staining, and liver/body weight ratio was assessed using a *t*-test or one-way ANOVA followed Tukey’s multiple comparison test wherever appropriate, with GraphPad Prism (GraphPad Software). Results were presented as mean with standard deviation (SD), considering *p*-values less than 0.05 as statistically significant. Investigators were not blinded to group allocation during the experiments or while evaluating results across all studies, including animal experiments. There was no estimate of variation within each group of data. Variance was similar among the groups being statistically compared. Kaplan-Meier survival analysis was employed to evaluate disease-free survival, with a log-rank test used to assess statistical significance.

## Supplementary information

supplementary information

uncropped western blots

## Data Availability

The scRNA sequencing data generated by this study has been deposited to GEO database under accession number (GSE302059). The mRNA levels of *ANXA1*, *S100A6*, *SOX2*, *STAT3*, *CD163* in human HCC patients were obtained from transcriptome sequencing data in The Cancer Genome Atlas (TCGA), accessed via the cBioPortal for Cancer Genomics (https://www.cbioportal.org/).
