# Supplementary material for: Tumor-derived annexin A1 drives lenvatinib resistance by regulating SOX2/STAT3/S100A6 feedback loop and M2 macrophage polarization in hepatocellular carcinoma
Source: Cell Death Dis. 2026 Jul 29;17(1):908. doi: 10.1038/s41419-026-09118-w (PMC13696695; doi:10.1038/s41419-026-09118-w)

Figure 2B

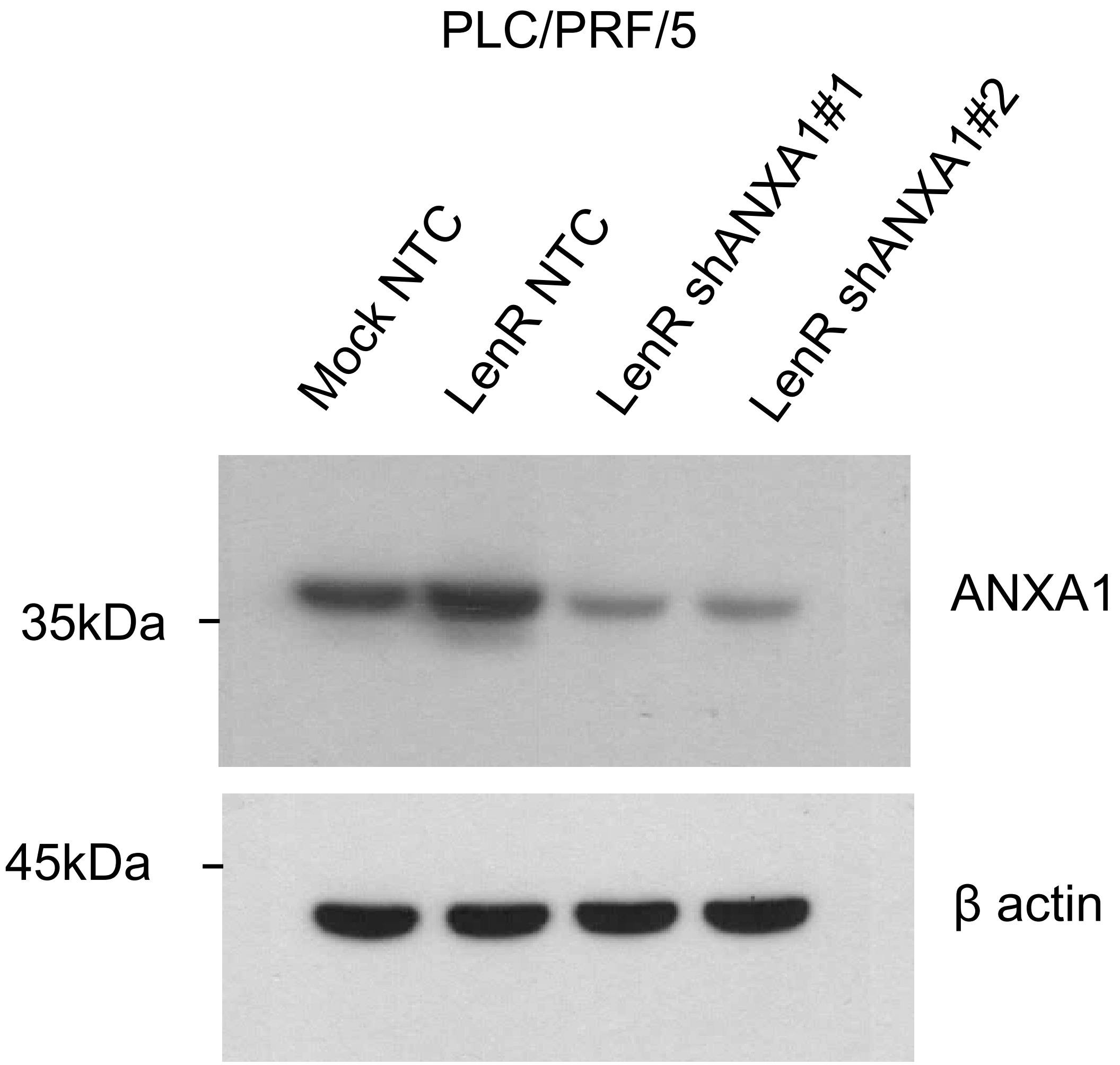
PLC/PRF/5
Mock NTC
LenR NTC
LenR shANXA1#1
LenR shANXA1#2
35kDa
ANXA1
45kDa
β actin

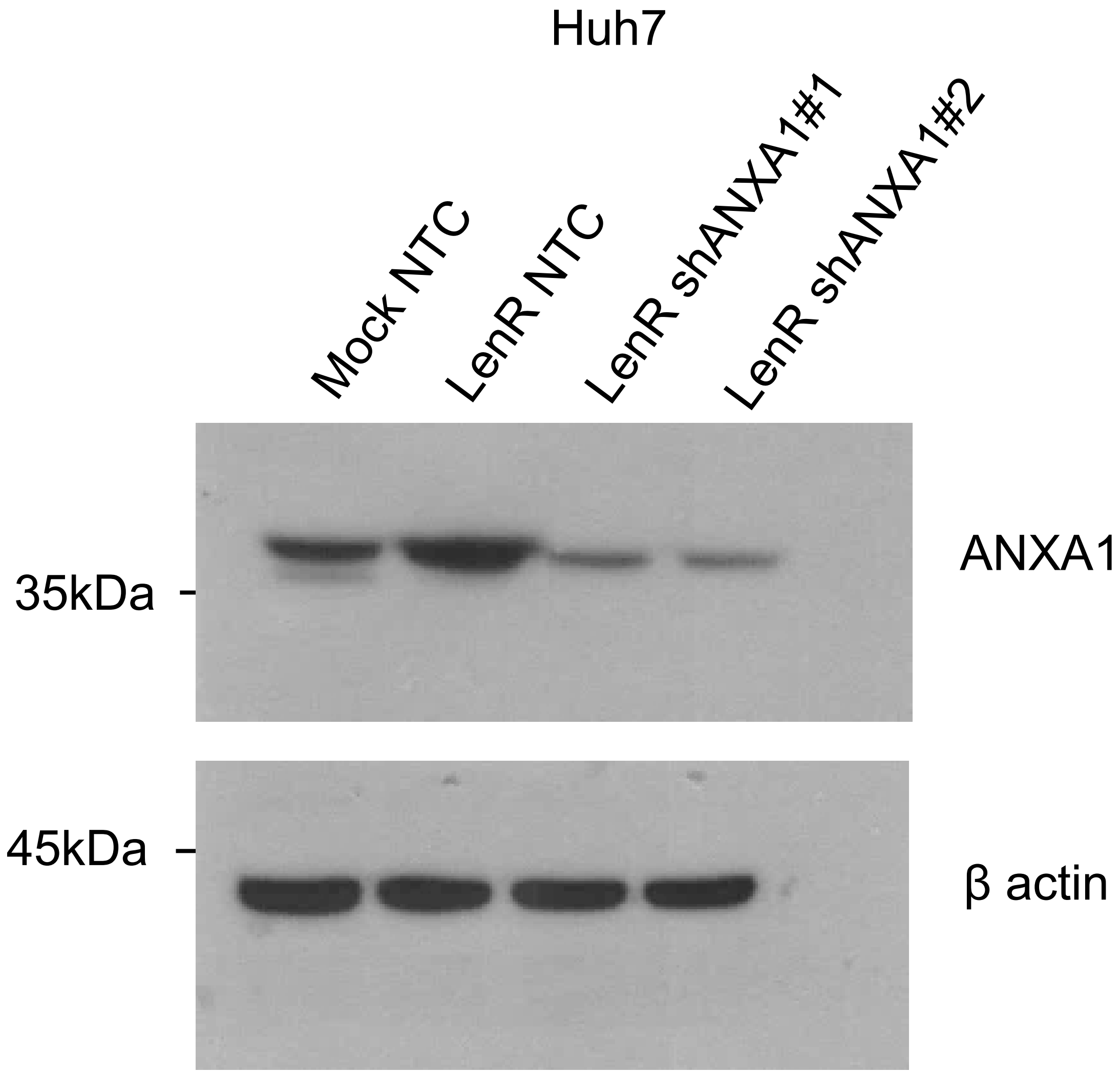
Huh7
Mock NTC
LenR NTC
LenR shANXA1#1
LenR shANXA1#2
ANXA1
35kDa
45kDa
β actin

Figure 3D

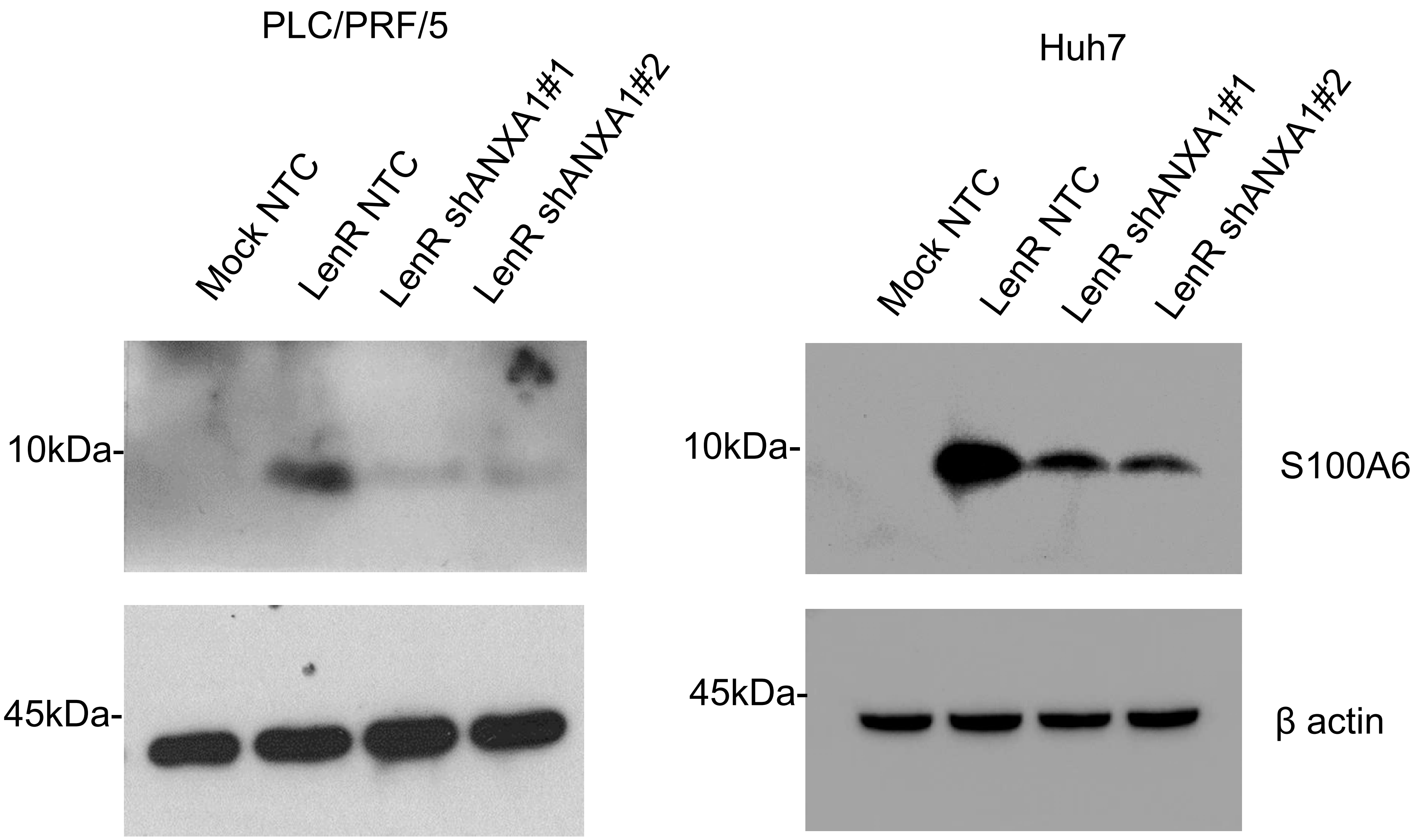
PLC/PRF/5
Mock NTC
LenR NTC
LenR shANXA1#1
LenR shANXA1#2
10kDa-
45kDa-
Huh7
Mock NTC
LenR NTC
LenR shANXA1#1
LenR shANXA1#2
10kDa-
45kDa-
S100A6
β actin

Figure 3E

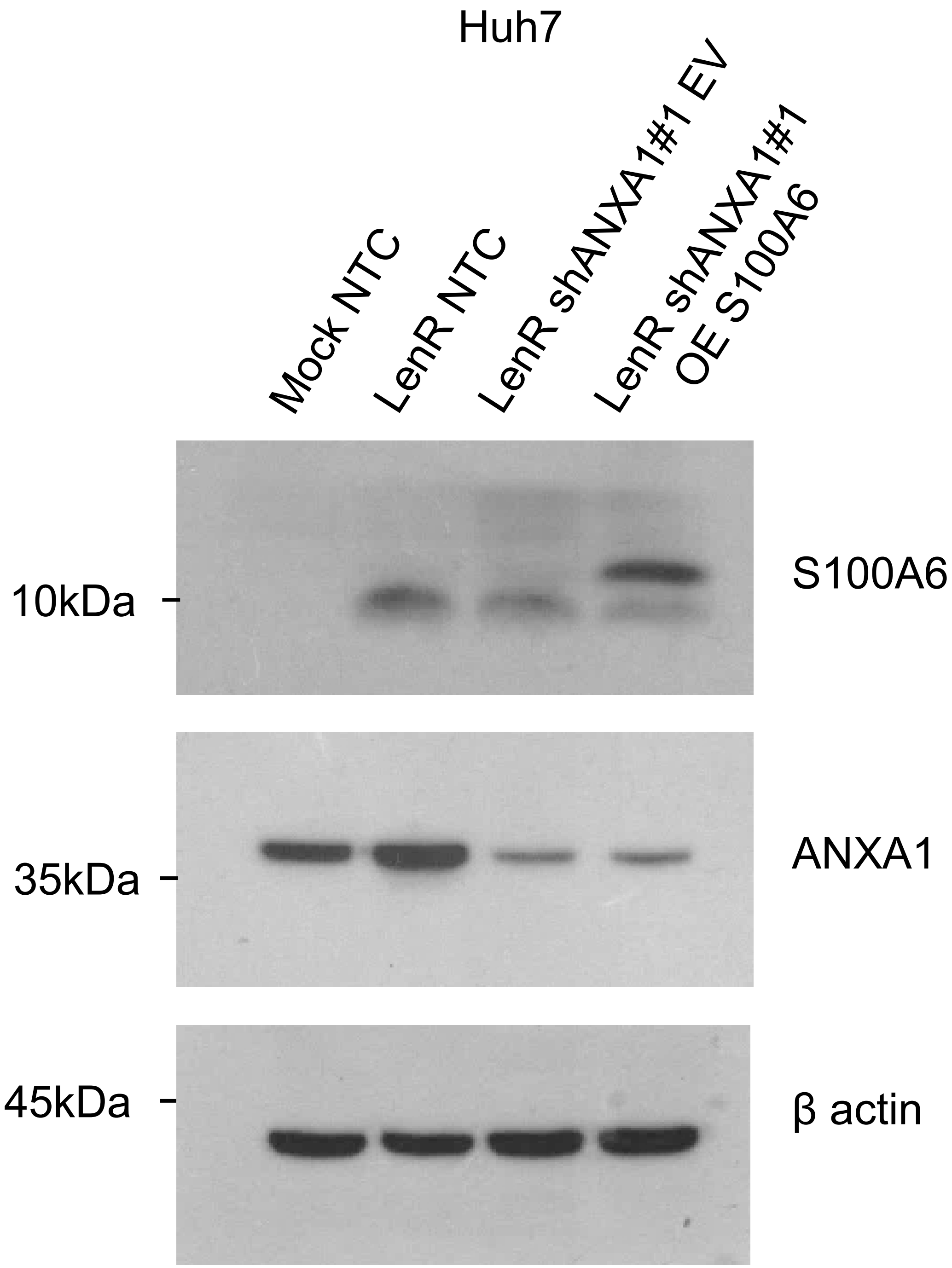

Huh7
Mock NTC
LenR NTC
LenR shANXA1#1 EV
LenR shANXA1#1
OE S100A6
10kDa
S100A6
35kDa
ANXA1
45kDa
β actin

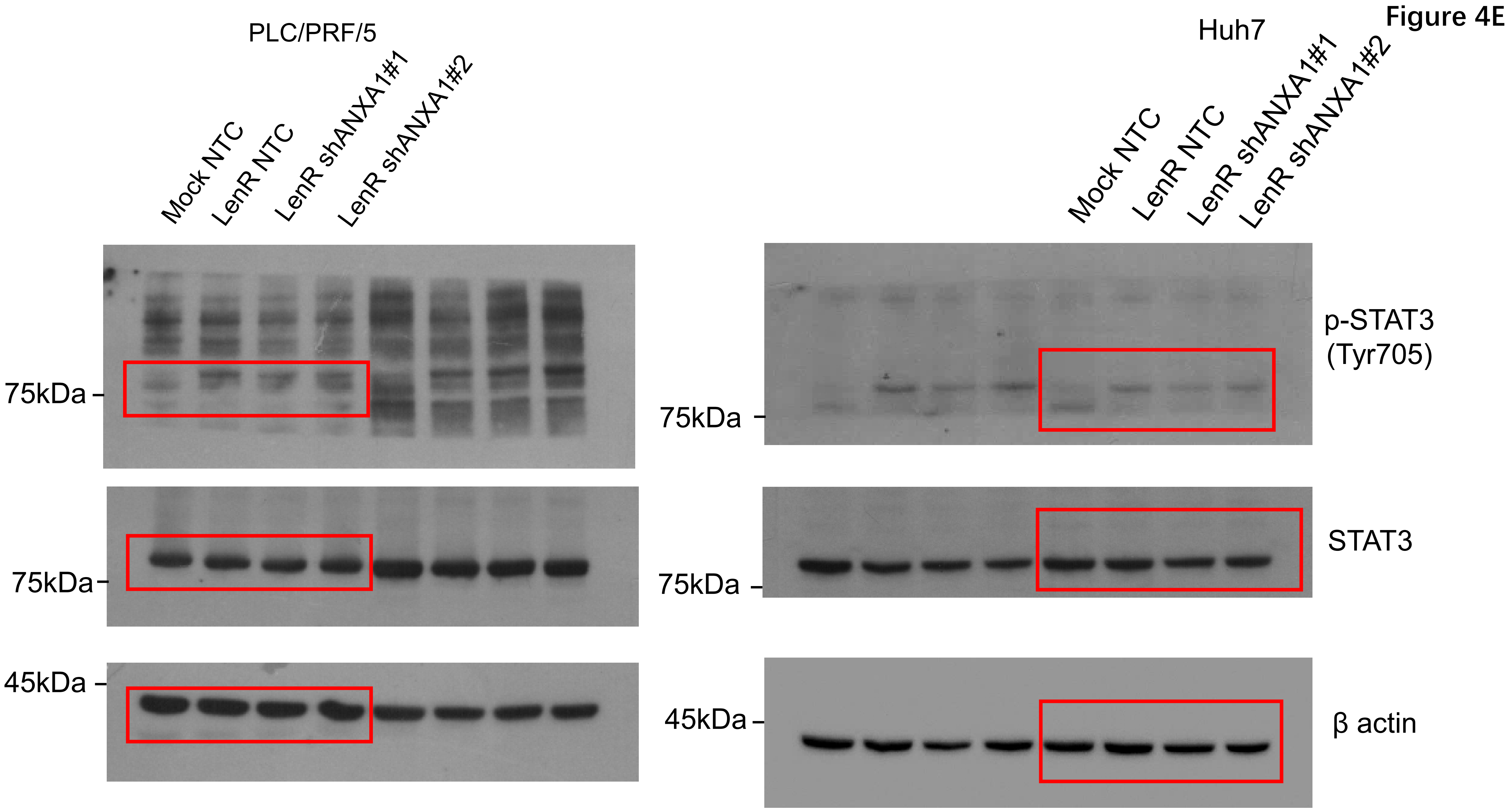

Figure 4E
PLC/PRF/5
Mock NTC
LenR NTC
LenR shANXA1#1
LenR shANXA1#2
Huh7
Mock NTC
LenR NTC
LenR shANXA1#1
LenR shANXA1#2
75kDa
75kDa
45kDa
75kDa
75kDa
45kDa
p-STAT3 (Tyr705)
STAT3
β actin

Figure 4F

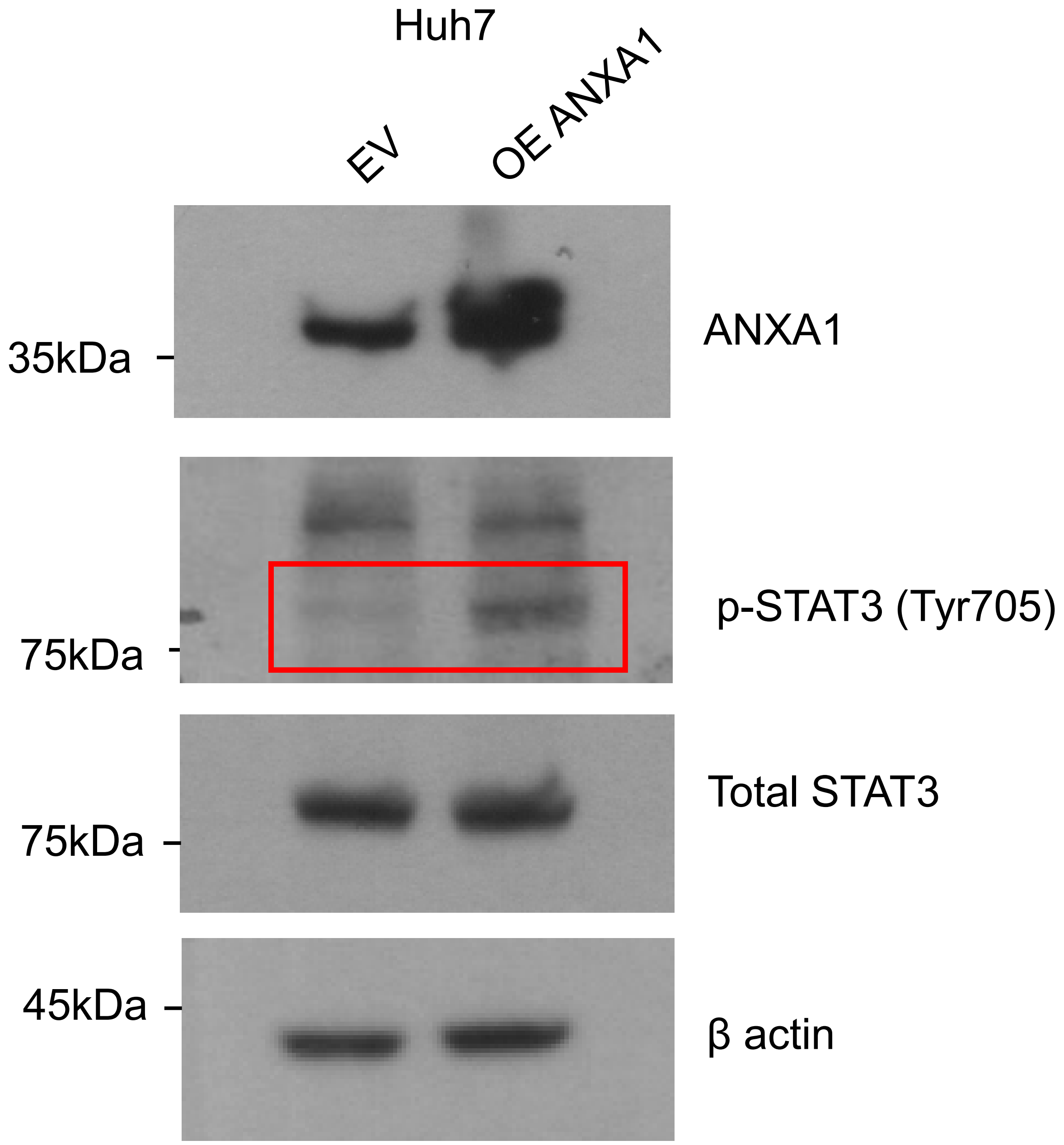
Huh7
EV
OE ANXA1
35kDa
ANXA1
75kDa
p-STAT3 (Tyr705)
75kDa
Total STAT3
45kDa
β actin

Figure 4G

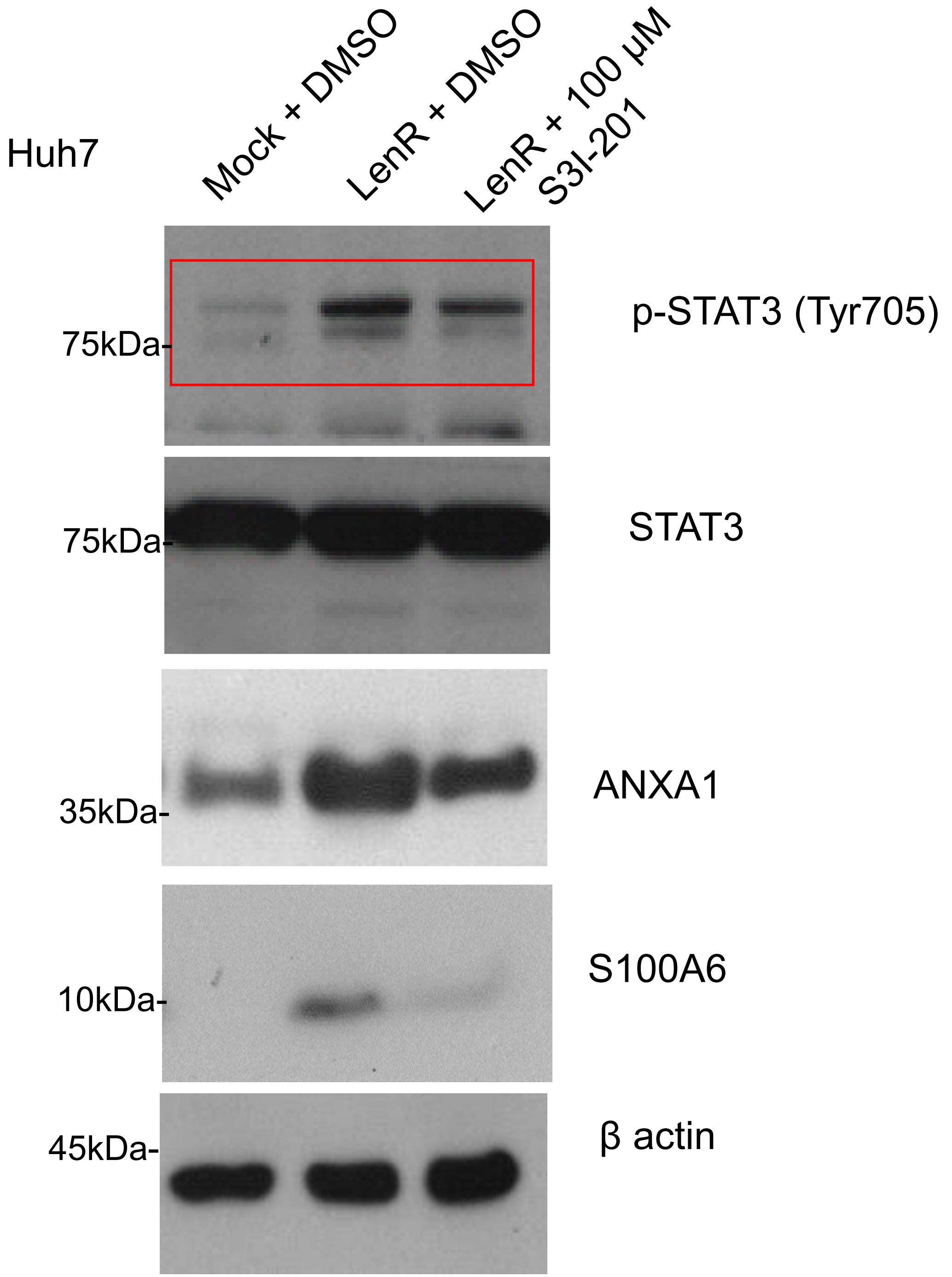

Huh7
Mock + DMSO
LenR + DMSO
LenR + 100 μM S3I-201
75kDa-
p-STAT3 (Tyr705)
75kDa-
STAT3
35kDa-
ANXA1
10kDa-
S100A6
45kDa-
β actin

Figure 5D

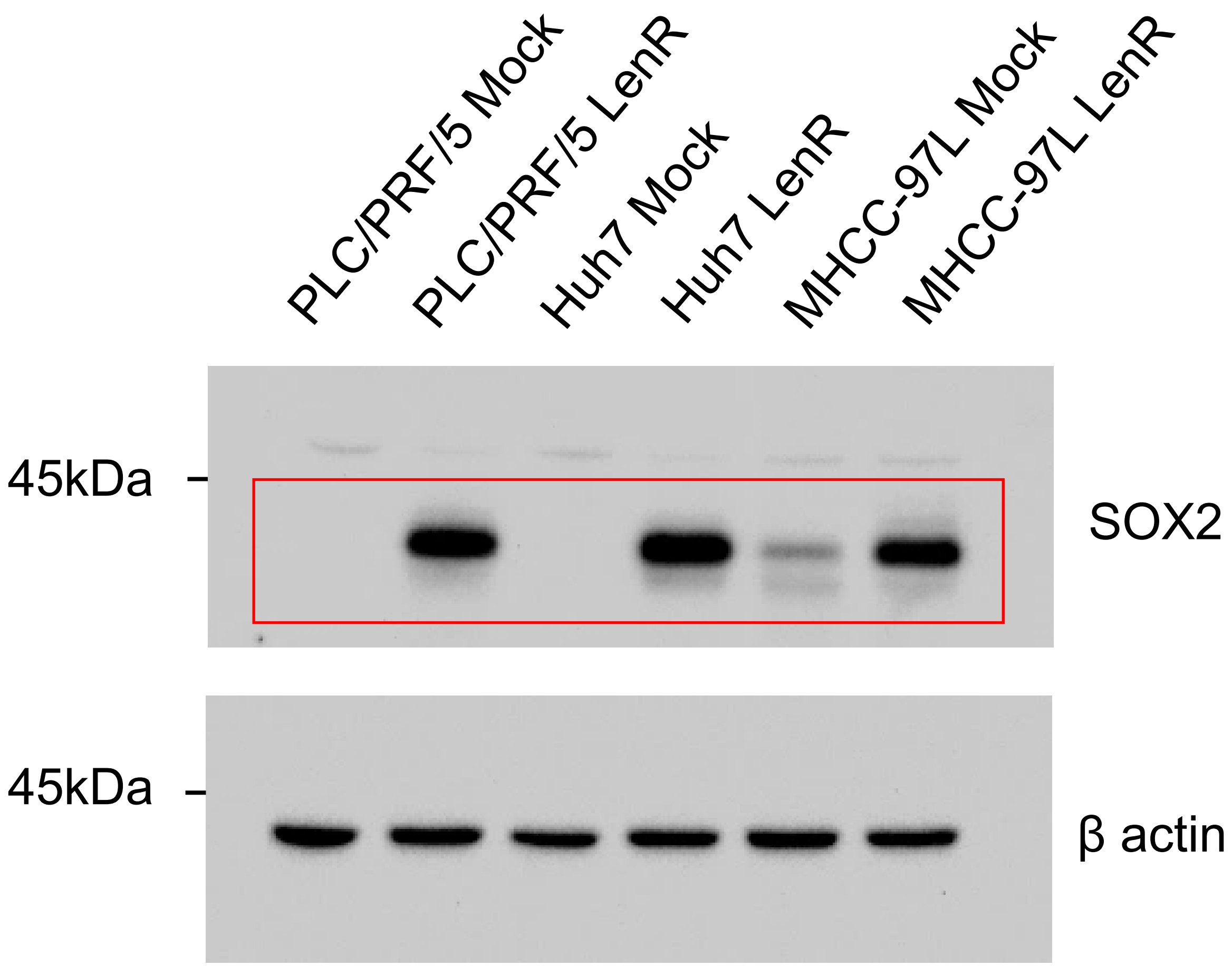
PLC/PRF/5 Mock
PLC/PRF/5 LenR
Huh7 Mock
Huh7 LenR
MHCC-97L Mock
MHCC-97L LenR
45kDa
SOX2
45kDa
β actin

Figure 5I

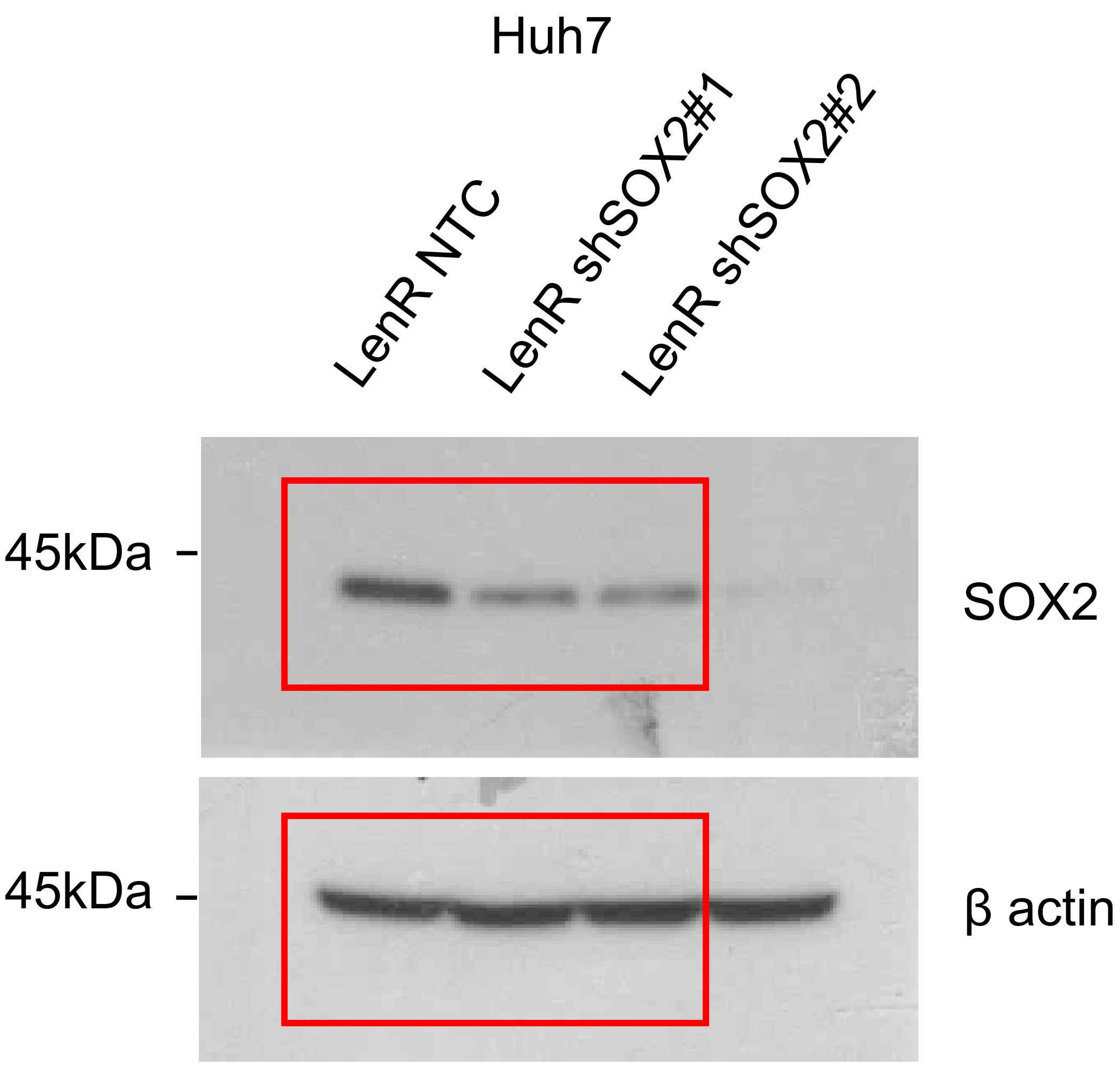
Huh7
LenR NTC
LenR shSOX2#1
LenR shSOX2#2
45kDa
SOX2
45kDa
β actin

Figure 6G

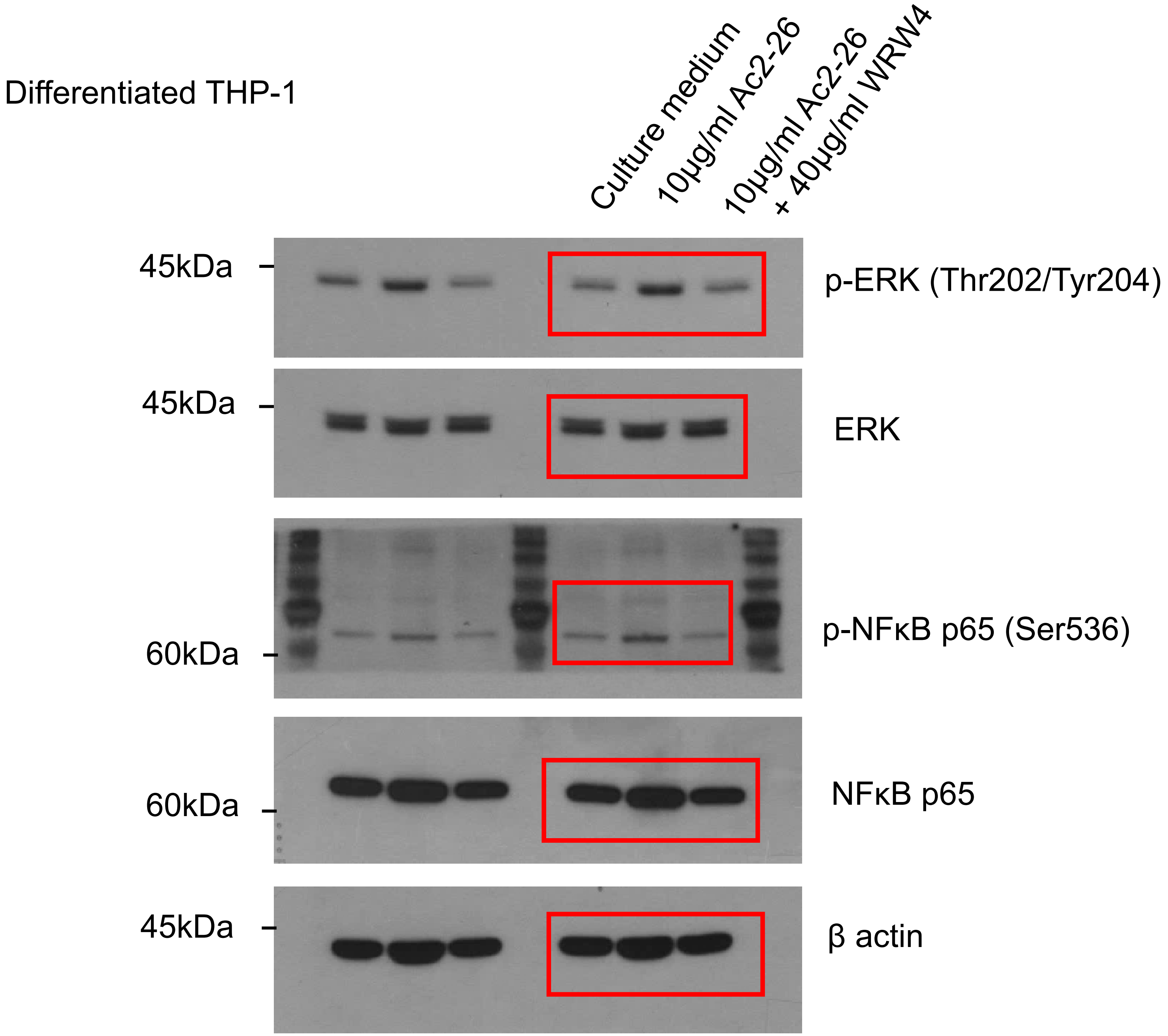
Differentiated THP-1
Culture medium
10µg/ml Ac2-26
10µg/ml Ac2-26
+ 40µg/ml WRW4
45kDa
p-ERK (Thr202/Tyr204)
45kDa
ERK
60kDa
p-NFκB p65 (Ser536)
60kDa
NFκB p65
45kDa
β actin

Supplementary Figure 1D

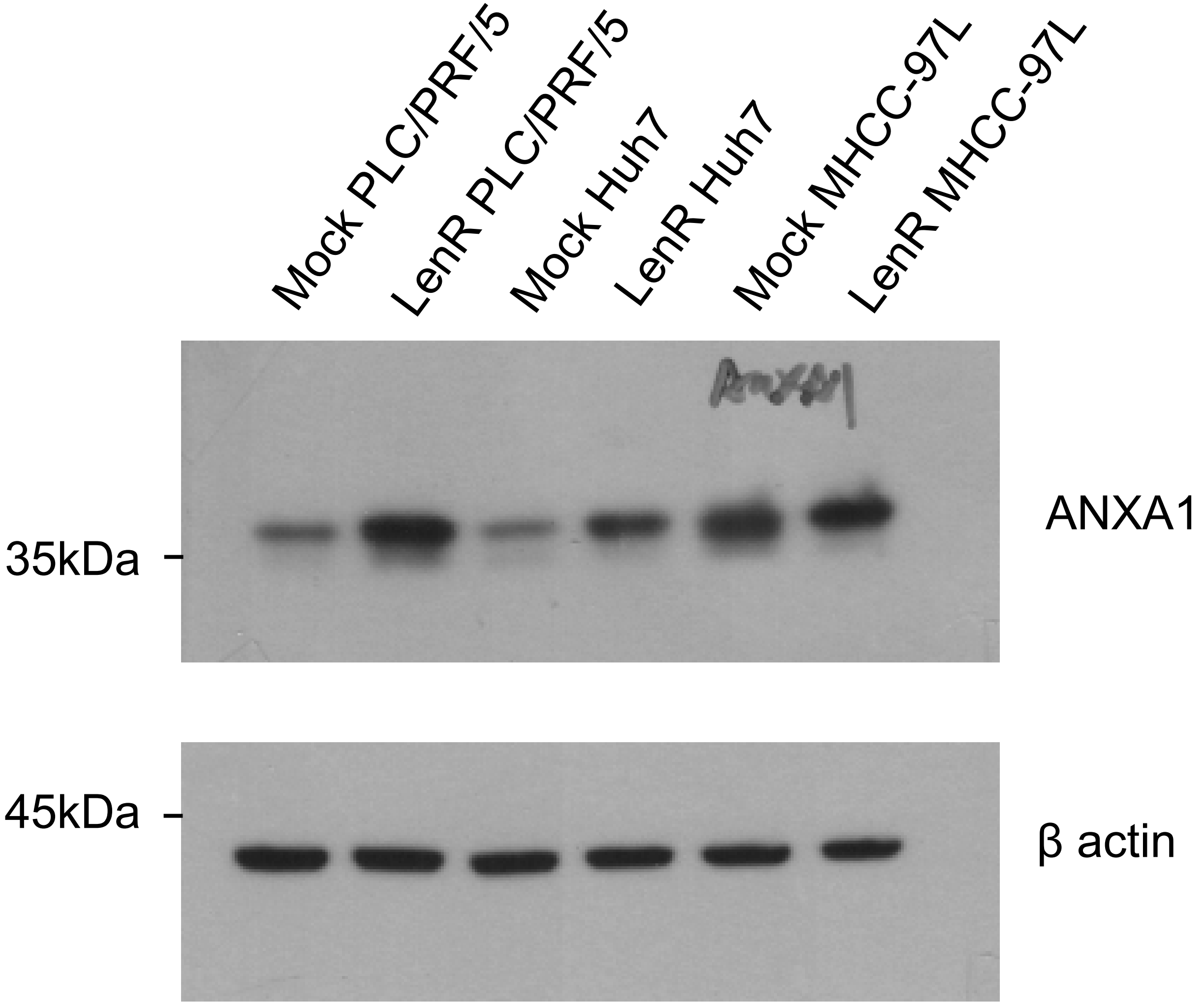

Supplementary Figure 5A

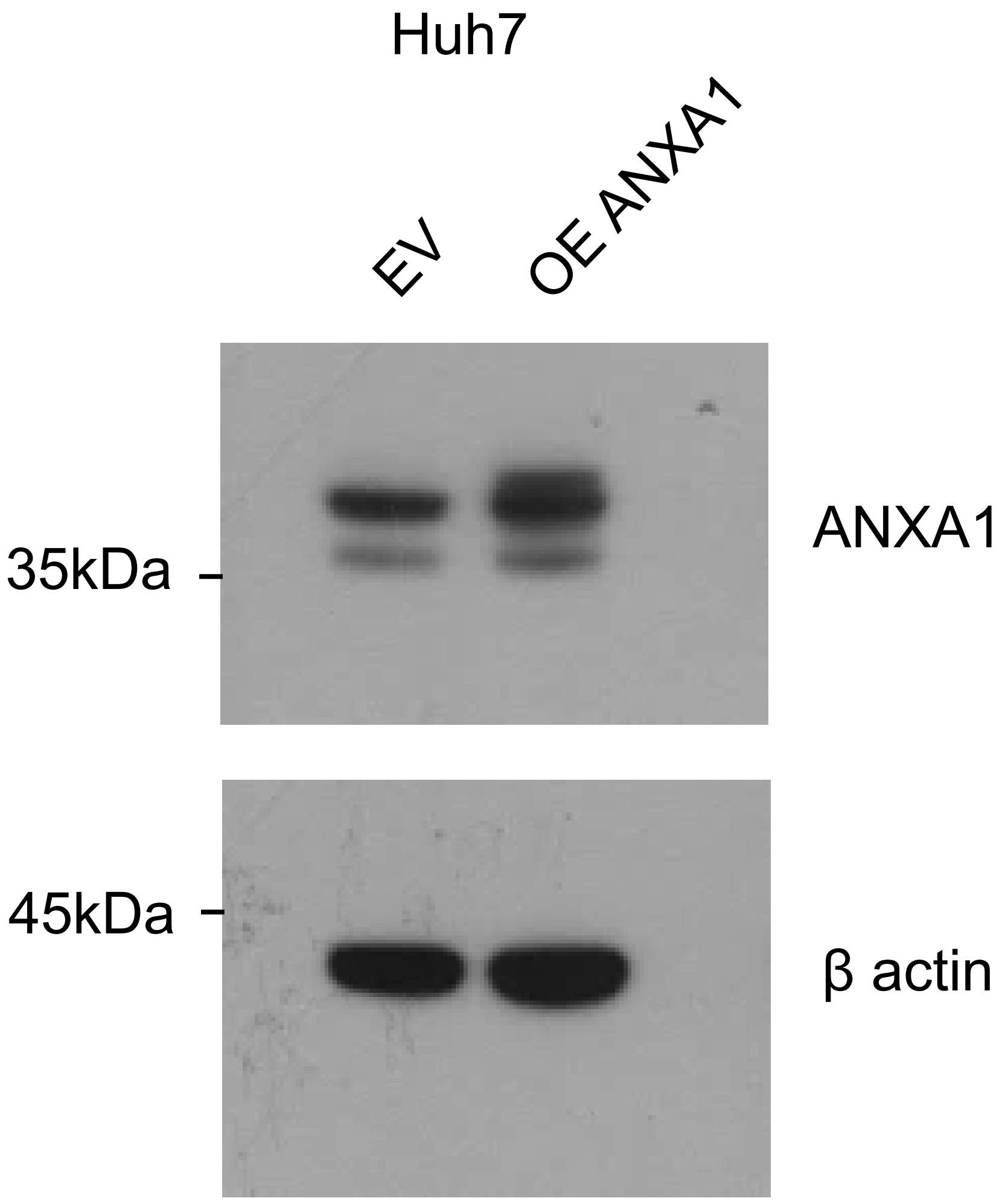

Supplementary Figure 7C

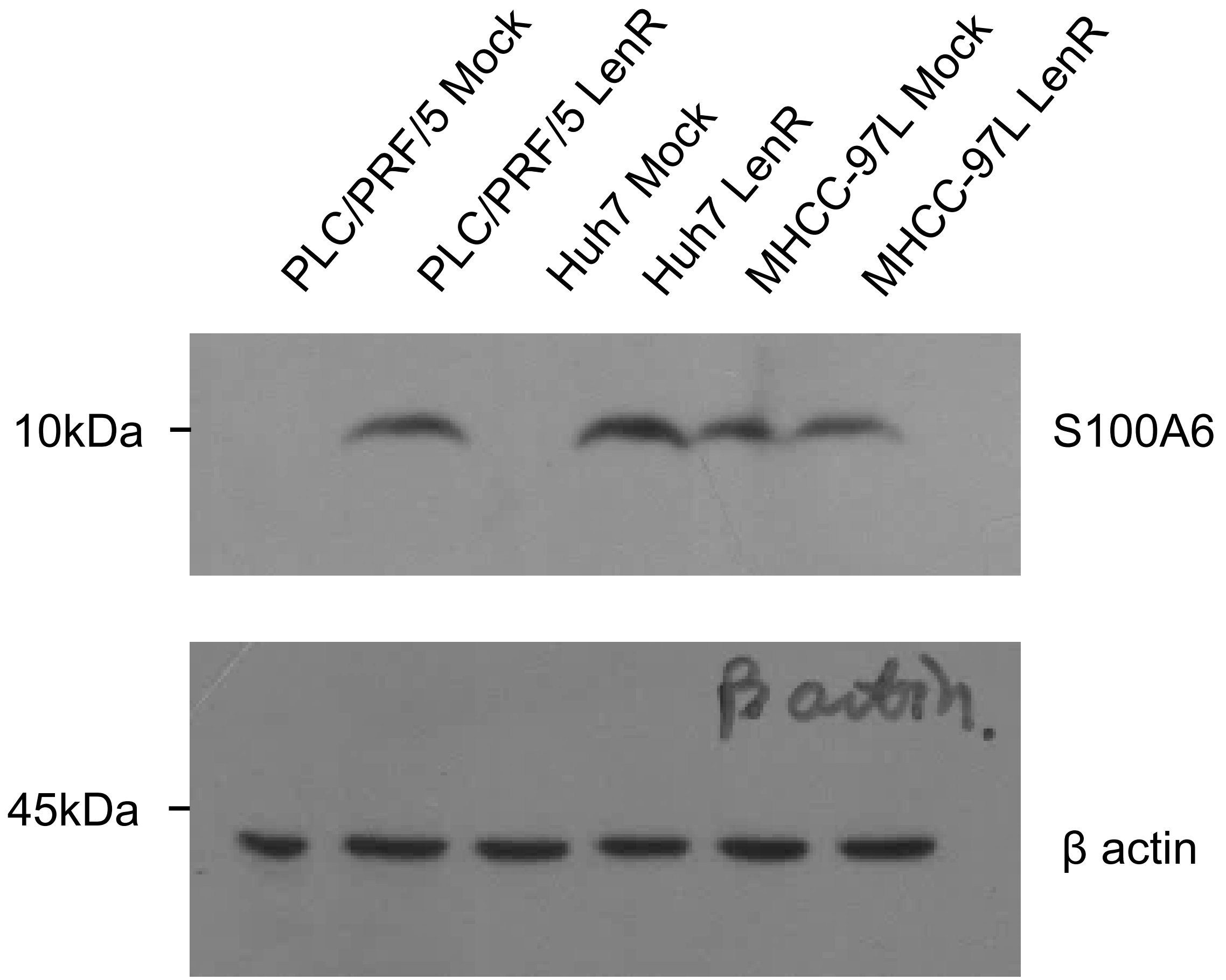

Supplementary Figure 8E

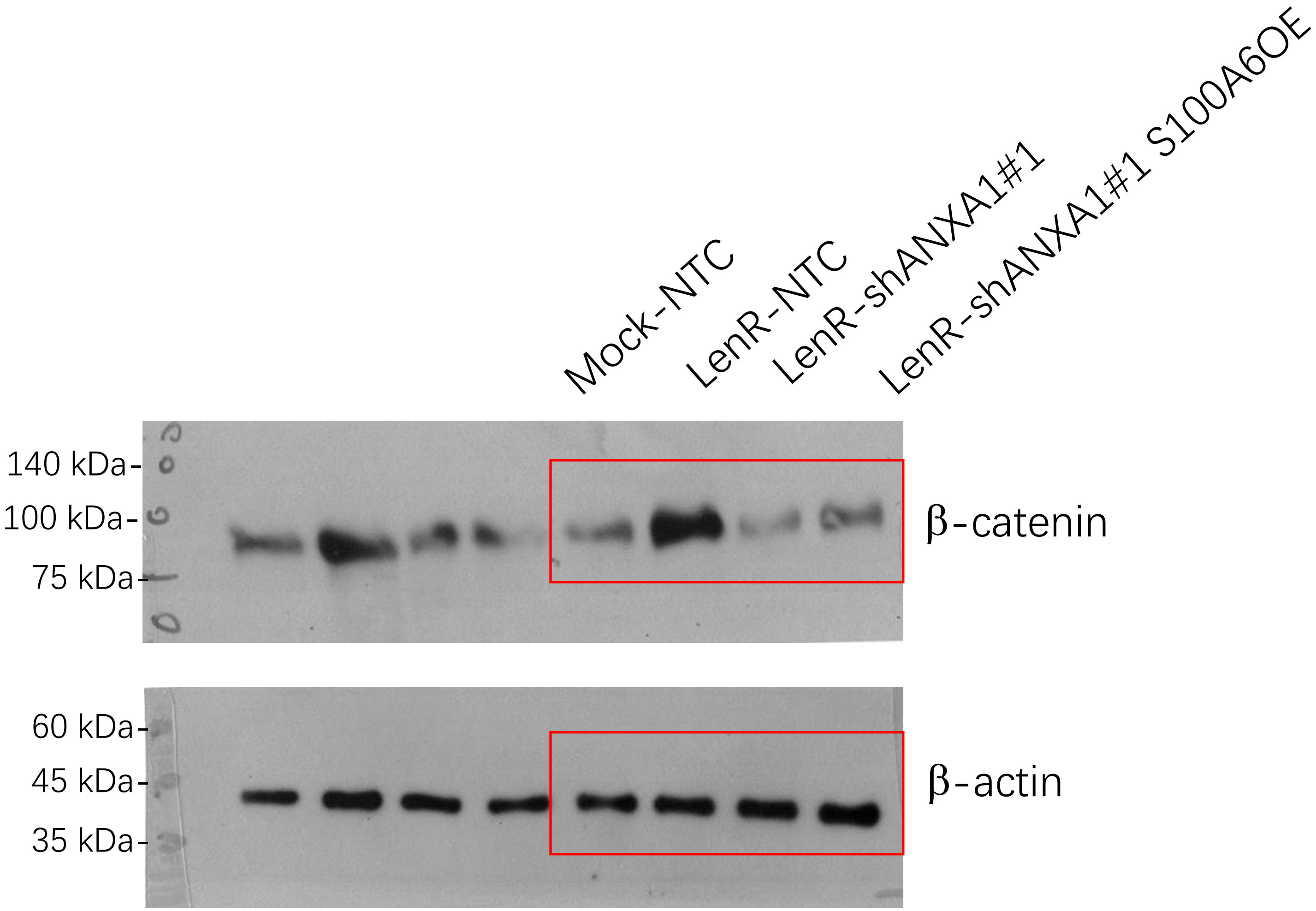

Supplementary Figure 9B

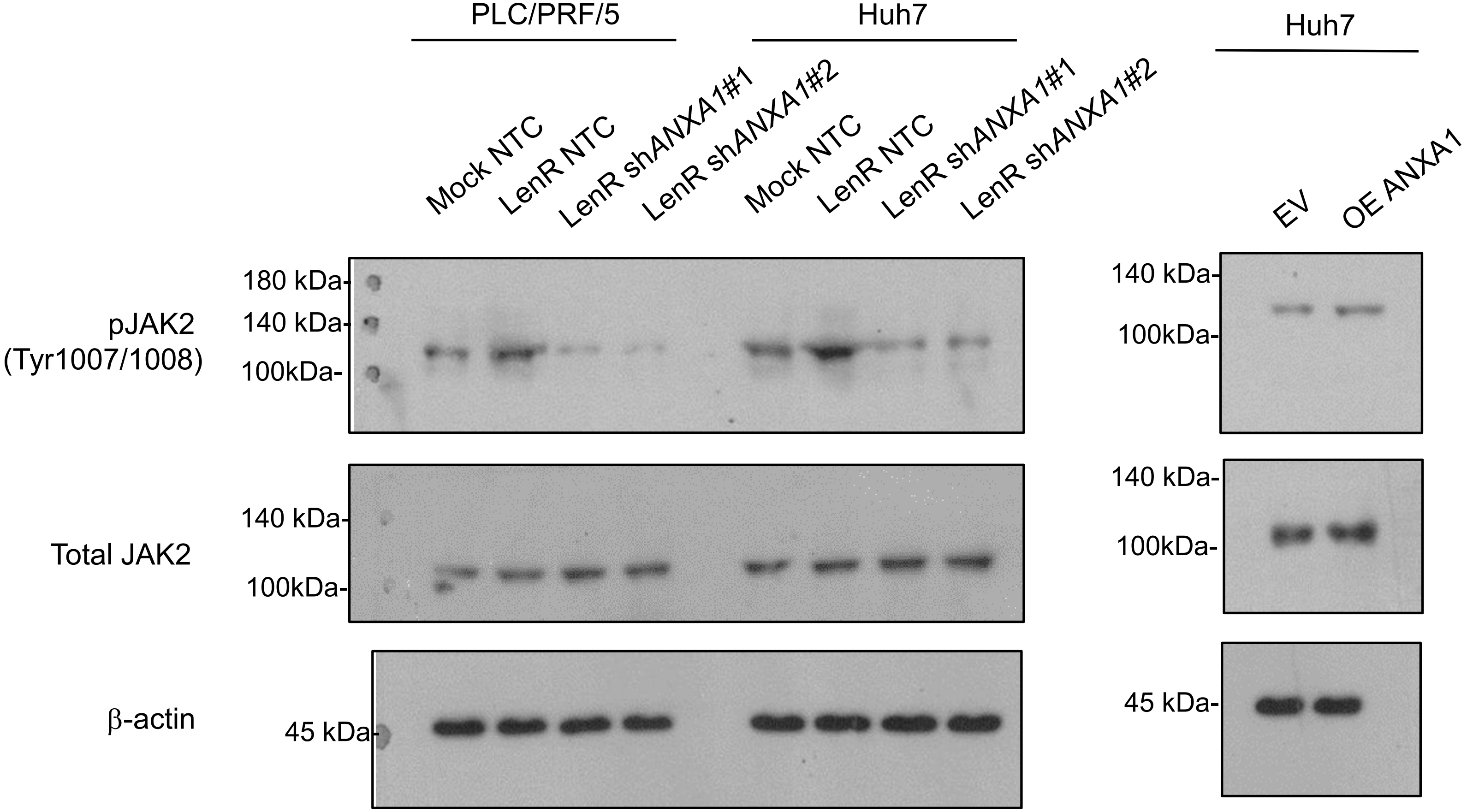

Supplementary Figure 10A

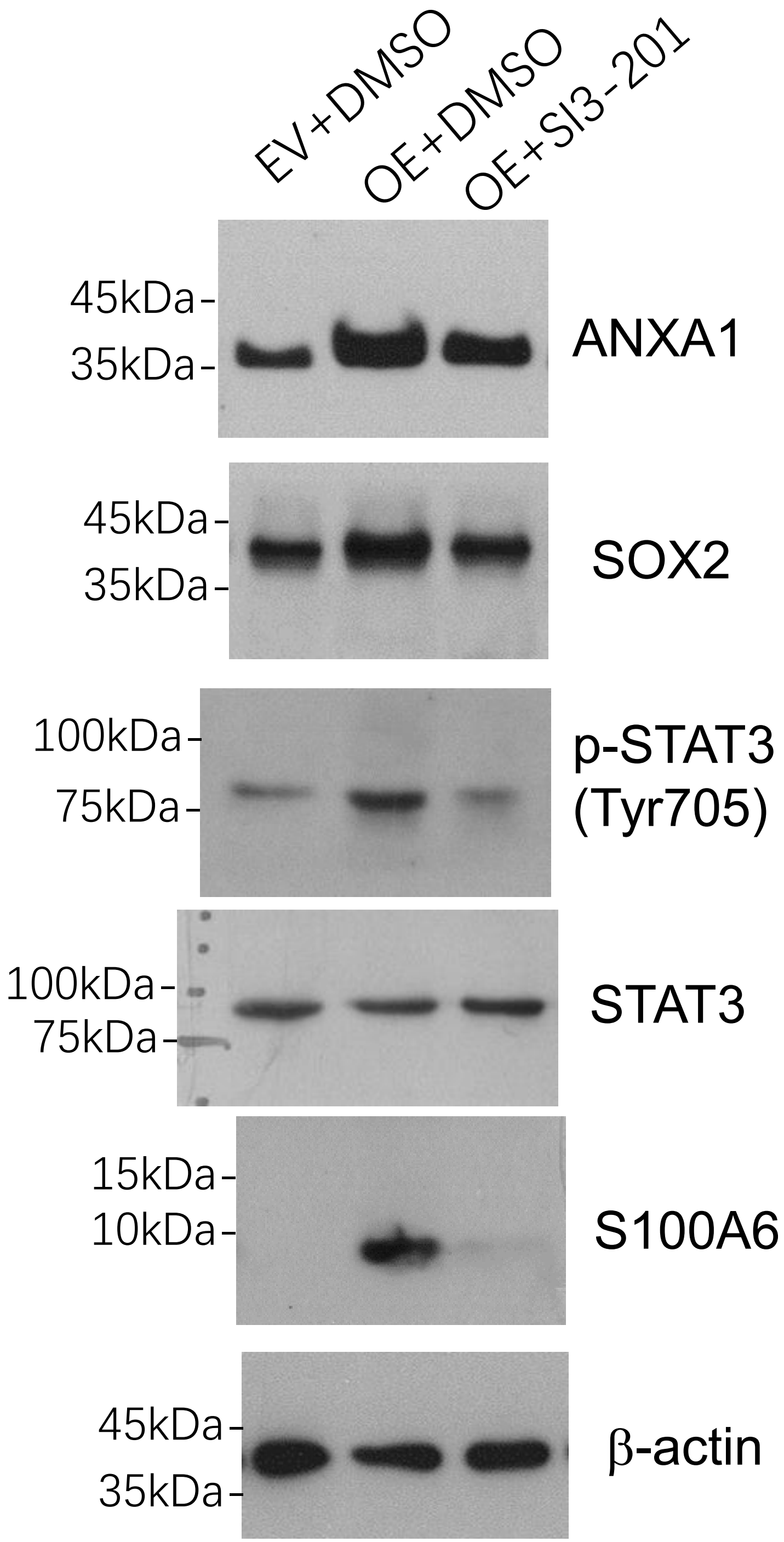

Supplementary Figure 12D

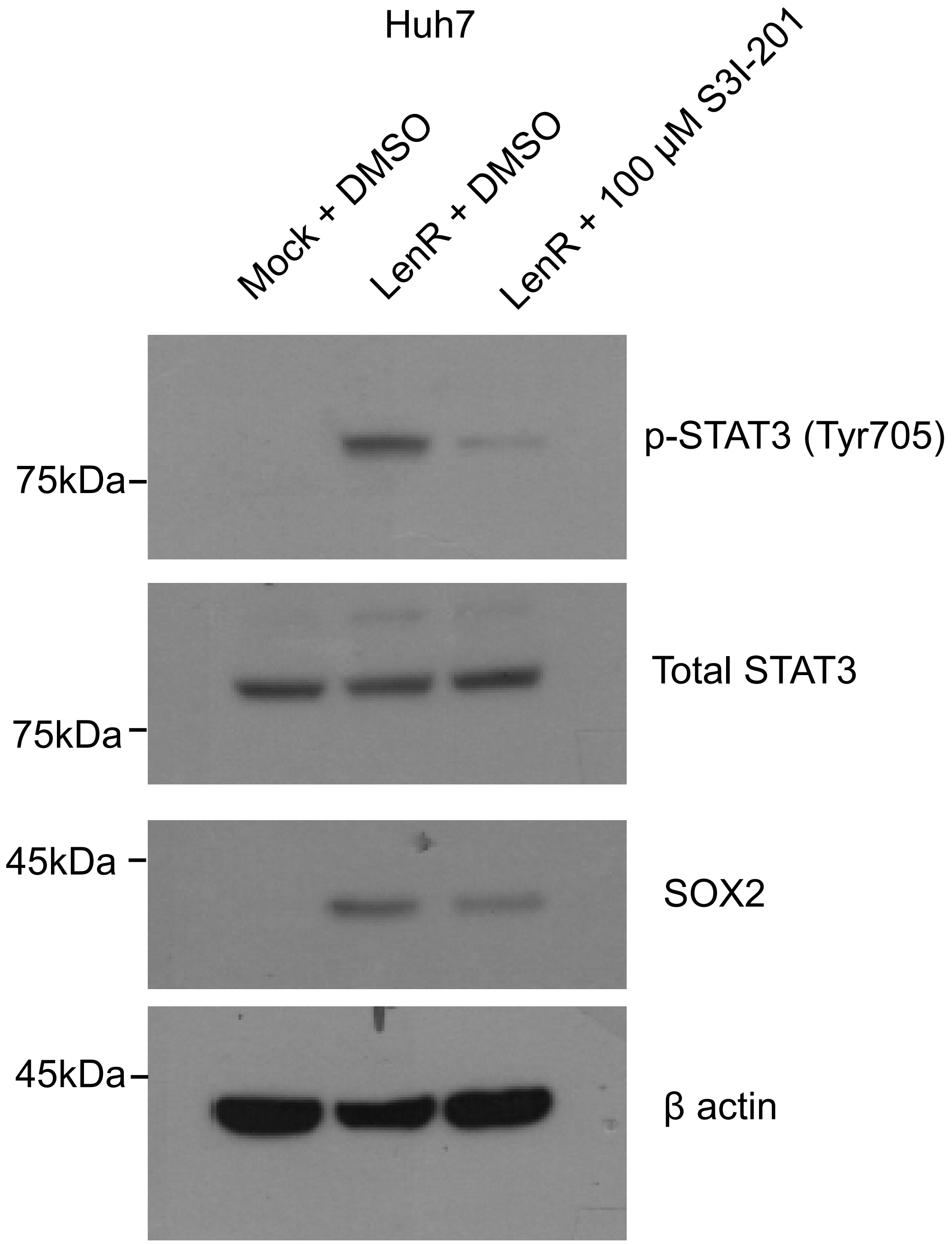

Supplementary Figure 12E

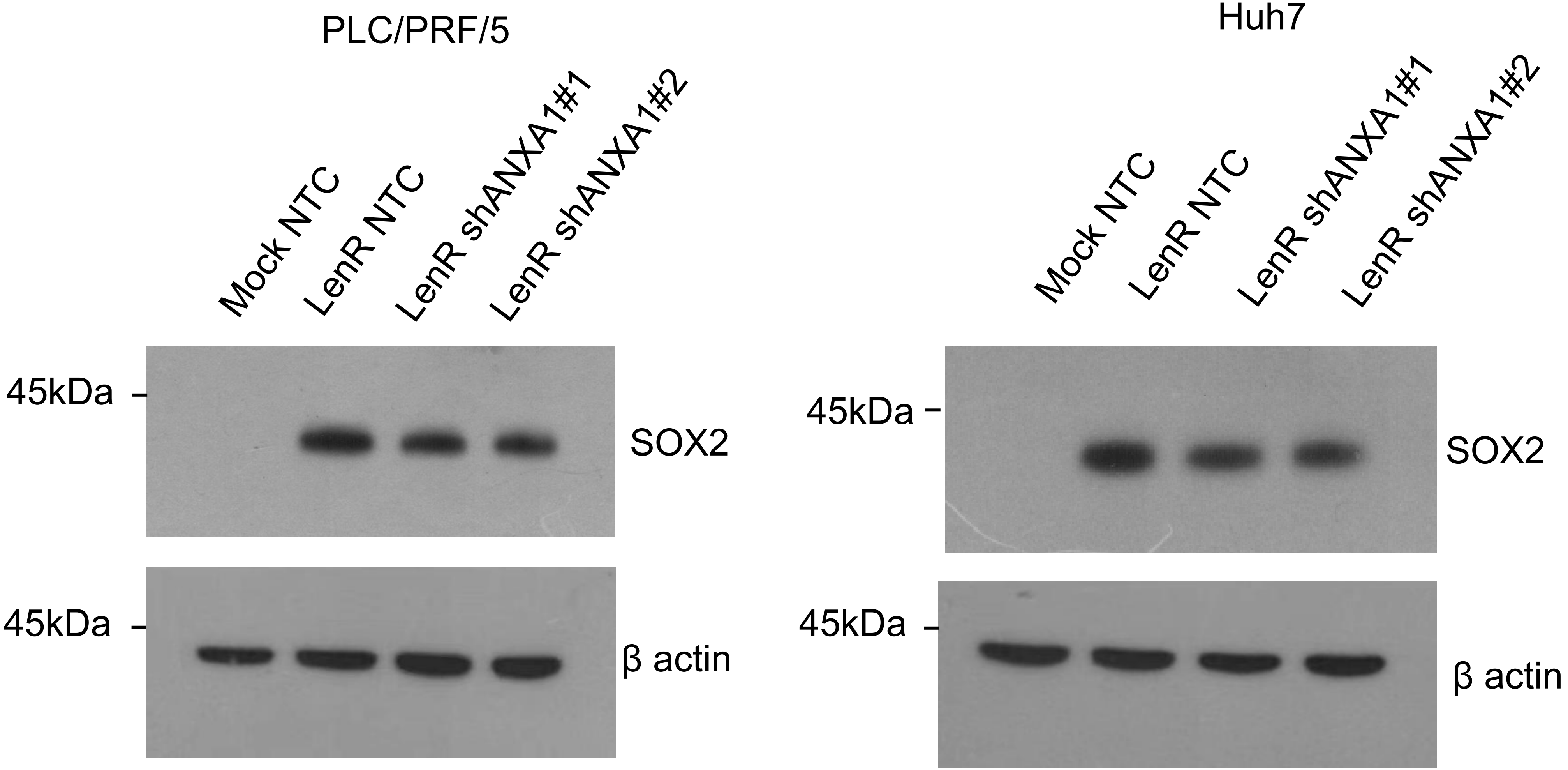

Supplement: Supplementary file 1 [file 41419_2026_9118_MOESM2_ESM.pdf]
