# Supplementary material for: Tumor-derived annexin A1 drives lenvatinib resistance by regulating SOX2/STAT3/S100A6 feedback loop and M2 macrophage polarization in hepatocellular carcinoma
Source: Cell Death Dis. 2026 Jul 29;17(1):908. doi: 10.1038/s41419-026-09118-w (PMC13696695; doi:10.1038/s41419-026-09118-w)
Supplement: Supplementary file 2 [file 41419_2026_9118_MOESM1_ESM.docx]

**Supplementary Information: Tumor-derived annexin A1 drives lenvatinib resistance by regulating SOX2/STAT3/S100A6 feedback loop and M2 macrophage polarization in hepatocellular carcinoma**

**Table of content:**

**Supplementary Materials and Methods**

**Supplementary Figure 1-18.**

**Supplementary Table 1. Clinicopathological features of the lenvatinib-sensitive and resistant HCC patients.**

**Supplementary Table 2. Effect of ANXA1 knockdown and overexpression on tumorigenicity of PLC/PRF/5 and Huh7 cells.**

**Supplementary Table 3. shRNA sequences for gene knockdown.**

**Supplementary Table 4. Primer sequences that target two predicted SOX2 binding sites in the ANXA1 promoter region for qPCR.**

**Supplementary Table 5. List of sequences of FL or truncated ANXA1 promoter.**

**Supplementary Table 6. Primer sequences for qRT-PCR.**

**Supplementary Materials and Methods**

**Reagents**

Lenvatinib was purchased from Selleckchem for *in vitro* experiments and from LC Laboratories for *in vivo* experiments. p-STAT3 inhibitor S3I-201 (#HY-15146) and FPR2 antagonist WRW4 (#HY-P1119) were purchased from MedchemExpress. ANXA1 N-terminal mimetic peptide Ac2-26 (#8045) was purchased from Tocris Bioscience.

**Cell lines and cell culture**

PLC/PRF/5 and Huh7 human HCC cell lines (Japan Cancer Research Bank), MHCC-97L human HCC cell line (Liver Cancer Institute, Fudan University), and 293FT (Invitrogen, Thermo Fisher Scientific) were cultured in Dulbecco’s Modified Eagle Medium (DMEM) with high glucose (4500mg/L) and L-glutamine (Gibco, Invitrogen) supplemented with 10% heat-inactivated fetal bovine serum (FBS) (Gibco, Invitrogen), 1% streptomycin and 1% penicillin (Gibco, Invitrogen) at 37°C in a humidified chamber with 5% CO_2_. THP-1 cells (a gift from Prof. Stephanie Ma, The University of Hong Kong) were maintained in RPMI1640 medium with 25 mM HEPES supplemented with 10% FBS, 0.05 mM β-mercaptoethanol, 1% streptomycin and 1% penicillin. Cells were passaged every 4 days. All cell lines used in this study were obtained between 2013 and 2016, and they were authenticated by morphological observation and STR DNA analysis with PowerPlex® 16HS kit (Promega) as well as tested for the absence of mycoplasma contamination (MycoAlert, Lonza). Cells were used within 20 passages after thawing.

**Establishment of LenR HCC cell clones**

LenR PLC/PRF/5, Huh7, and MHCC-97L cells were developed through continuous administration of lenvatinib, with the concentration gradually increased to 30 µM over a period of 6 months. An equivalent concentration of DMSO was used as a mock control. The resulting LenR cell clones were maintained in complete medium supplemented with 10 µM lenvatinib.

**Knockdown of ANXA1 and SOX2 expression**

To knockdown ANXA1 in human HCC cells, two different short hairpin (shRNA) sequences against ANXA1 were cloned to pLKO.1-puro plasmid (Addgene). 293FT cells were transfected with the plasmids using lipofectamine2000 (Invitrogen) and packaging plasmid mix. Viral supernatant collected from 293FT cells was used to infect mock and LenR PLC/PRF/5 and Huh7. The stable knockdown cell clones were generated by puromycin selection. The establishment of SOX2-knockdown stable cell clones were used the same method. The shRNA sequences were listed in **Supplementary Table 3**.

**Overexpression of ANXA1 and S100A6**

To overexpress ANXA1 expression in human HCC cells, ANXA1 open reading frame (ORF) expression clone (#EX-A88517-Lv158, GeneCopoeia) was transfected into 293T cells using Lipofectamine2000. Viral supernatant collected from 293T cells was used to infect Huh7 cells. The stable ANXA1 overexpression cell clones were generated by neomycin selection. Overexpression of S100A6 was established via transfecting LenR sh*ANXA1*#1 PLC/PRF/5 and Huh7 cells by S100A6 human Myc-DDK-tagged ORF clone (#RC203951, ORIGENE) using Lipofectamine2000.

**Limiting dilution assay**

Human HCC cells were seeded into a 96-well plate pre-coated with polyHEMA in a serial dilution manner using DMEM/F12 medium containing 0.25% methylcellulose. Sphere formation was scored under a phase contrast microscope. The frequency of spheroid-forming stem cells was calculated using extreme limiting dilution analysis (ELDA) software ^1^.

**RNA extraction and real-time quantitative PCR (RT-qPCR) analysis**

Total RNA was extracted using TRIzol reagent according to the manufacturer’s instructions (Invitrogen). Complementary DNA (cDNA) was synthesized from purified RNA using PrimeScript RT Reagent Kit (Takara Bio, Shiga). The procedures of cDNA synthesis followed the manufacturer’s protocol. The synthesized cDNA was then subjected to qPCR with BrightGreen 2X qPCR Master Mix (Applied Biological Materials Inc, Richmond) using QuantStudio™ 7 Flex Real-Time PCR System (Applied Biosystems) with primers specific to the sequences of genes of interests which were listed in **Supplementary Table 6**. Relative mRNA expression fold change differences were calculated using 2^−ΔΔCT^ method with the references to human HPRT.

**ChIP-qPCR assay**

ChIP was performed using Magna ChIP^TM^ G Kit (#MAGNA0002, Merck Millipore) according to the manufacturer’s instructions. DNA-protein complexes were sheared using Sonifier (#SFX550, BRANSON) and immunoprecipitated overnight using Magna ChIP^TM^ G (#17-611, Merck Millipore) and SOX2 antibody (#23064S, Cell Signalling Technology) or normal IgG antibody (#2729P, Cell Signalling Technology) as the control. DNA was purified using QIAquick PCR Purification Kit (#28106, QIAGEN). The purified DNA was subject to qPCR with BrightGreen 2X qPCR Master Mix (Applied Biological Materials Inc, Richmond) using QuantStudio™ 7 Flex Real-Time PCR System (Applied Biosystems) with primers that specifically targeted predicted SOX2 binding sites that were listed in **Supplementary Table 4**. The signals obtained were expressed as a percent of the total input chromatin: 2% x 2^(C[T] 2%input - C[T] IP Sample)^.

**Luciferase reporter assay**

Truncated or Full Length (FL) ANXA1 promoter region was cloned into pGL3-Basic Firefly luciferase reporter vector (#E1751, Promega). LenR PLC/PRF/5 cells and Huh7 cells were co-transfected with pRL-CMV Renilla luciferase control reporter vector (#E2261, Promega) using Lipofectamine2000. Luciferase activities were measured using Dual-Luciferase Reporter Assay System (#E1910, Promega) according to manufacturer’s protocol. The cloned FL or truncated ANXA1 promoter sequences were listed in the **Supplementary Table 5**.

**Enzyme-linked immunosorbent assay (ELISA)**

The CM from human HCC cells were collected after 72h incubation in serum free medium and centrifuge at 3000 x g for 10 minutes. The concentration of ANXA1 in the CM was detected using Human Annexin A1 ELISA kit (#ab222868, Abcam) according to manufacturer’s protocol.

**Annexin V apoptosis assay**

cells were stained with FITC-conjugated Annexin V (BioVision) and propidium iodide (PI) (Invitrogen) mixed in Annexin V binding buffer (BD Biosciences) for 30 minutes at room temperature after 48 hours under 40 µM lenvatinib treatment. Apoptosis percentage was detected using BD Accuri C6 flow cytometer (BD Biosciences) and was analyzed in FlowJo software.

**Western blot analysis**

Total protein from human HCC cell lines was extracted using RIPA lysis buffer supplemented with phosphatase inhibitor (Roche) and protease inhibitor (Roche). Protein concentration was determined using Bradford assay. Protein lysate was separated based on protein molecular weight using sodium dodecyl sulfate-polyacrylamide gel electrophoresis (SDS-PAGE) and transferred onto polyvinylidene difluoride (PVDF) membrane (Millipore) by electroblotting. The membrane was then blocked with 5% milk in Tris-buffered saline with 0.1% Tween® 20 detergent (TBST). Primary antibodies being used were ANXA1 (1:10000, #ab214486, Abcam), S100A6 (1:1000, #10245-1-AP, Proteintech), SOX2 (1:2000, #23064S, Cell Signalling Technology), p-STAT3 (Tyr705) (1:1000, #9145S, Cell Signalling Technology), STAT3 (1:1000, #12040S, Cell Signalling Technology), p-NF-κB p65 (Ser536) (1:1000, #3036S, Cell Signalling Technology), NF-κB p65 (1:1000, #sc-372, Santa Cruz Biotechnology), p-ERK (T202/Y204) (1:1000, #9101S, Cell Signalling Technology), ERK (1:1000, #9102S, Cell Signalling Technology), β-catenin (1:1000, #8480S, Cell Signalling Technology), pJAK2 (Tyr1007/1008) (1:1000, #3771, Cell Signalling Technology), JAK2 (1:1000, #3230, Cell Signalling Technology) and β-actin (1:5000, #A5316, Sigma-Aldrich). After overnight incubation with primary antibodies at 4°C, membrane was washed with TBST for 30 minutes. Anti-mouse or anti-rabbit secondary antibody conjugated with horseradish peroxidase (GE HealthCare) was applied to the membrane and incubated for 1h at room temperature. Membrane was then washed with TBST for 1-2 hours. The signals were detected using enhanced chemiluminescence method (WesternBright^TM^ ECL, advansta). Western blot images were quantified by densitometry using ImageJ software.

***In vitro* macrophage polarization**

THP-1 cells were seeded into 6-well tissue culture plates at the density of 5 x 10^5^ cells/ml containing 100 nM phorbol 12-myristate 13-acetate (PMA) (#P8139, Sigma-Aldrich) for inducing the differentiation of THP-1 cells towards M0 macrophage state. After 24 hours, the culture medium containing 100 nM PMA was withdrawn and complete culture medium was added to let cells undergo a 24h resting period to develop M0 macrophages. Fresh culture supernatant from 80% confluent human HCC cell lines was then added to M0 macrophages to induce macrophage polarization for 72 hours. In addition, 10 µg/ml Ac2-26 and 40 µg/ml WRW4 was added to M0 macrophages to examine their polarization conditions.

**Flow cytometry**

Cells were incubated with fluorochrome-conjugated antibody CD163-PE (#326505, BioLegend), CD86-PE (#12086942, eBioscience) or isotype control IgG. The percentage of CD163^+^ and CD86^+^ cells were detected using BD Accuri C6 flow cytometer (BD Biosciences) and was analyzed in FlowJo software.

**Immunofluorescence staining**

Human HCC cells were seeded on glass coverslips in 6-well tissue culture plates, fixed in 4% paraformaldehyde in phosphate buffered saline (PBS) for 15 minutes, and permeabilized with 0.1% Triton-X100 for 15 minutes. Cells were then blocked with 5% bovine serum albumin (BSA) in TBST for 1 hour. Successively, the cells were incubated with primary antibody ANXA1 (1:500, #ab214486, Abcam) or normal IgG antibody (1:500, #2729P, Cell Signalling Technology) at 4°C overnight. Anti-rabbit secondary antibody conjugated with Alexa Fluor^TM^ 488 (1:500, #A-11008, Invitrogen) were added for one hour of incubation at room temperature. Slides were counterstained with DAPI (Invitrogen) for nuclei, mounted with ProLong^TM^ Gold Antifade Mountant and examined by Leica TCS SPE confocal microscope.

**Immunohistochemical analysis of HCC specimens and resected mouse tumors**

Tissue sections were deparaffinized in xylene (BIOGNOST) and rehydrated in 100% ethanol. The sections were subject to antigen retrieval by a standard microwave heating technique either using diluted 50x Envision FLEX Target Retrieval Buffer (pH9, #K8004, DAKO) for ANXA1, SOX2, and CD163, or diluted 50x Envision FLEX Target Retrieval Buffer (pH6, # K8005, DAKO) for S100A6 and p-STAT3 (Tyr705). 3% hydrogen peroxide solution was applied to inhibit endogenous peroxidase activities. The sections were immersed in serum-free protein block (DAKO) and were subsequently incubated with primary antibody (ANXA1, 1:4000, #ab214486, Abcam; S100A6, 1:800, #10245-1-AP, Proteintech; p-STAT3 (Tyr705), 1:100, #9131S, Cell Signalling Technology; SOX2, 1:250, #23064S, Cell Signalling Technology; CD163, 1:500, #ab182422, Abcam). The sections were washed in TBST and incubated with anti-rabbit/mouse REAL EnVision^TM^ HRP-conjugated secondary antibody (DAKO). Positive staining was detected by EnVision™ FLEX DAB+ Substrate-Chromogen System (DAKO). The sections were counterstained with haematoxylin and Scott’s solution (BIOGNOST). The slides were mounted with BioMount solution (BIOGNOST) and examined using light microscope. The protein expression was quantified using Fiji ImageJ software.

**Multiplexed fluorescent immunohistochemistry in HCC and mouse tumors**

The Opal 4-Color Manual IHC Kit (#NEL810001KT, Akoya Biosciences) was used for multiplexed IHC staining. Tissue sections were deparaffinized in xylene (BIOGNOST) and rehydrated in 100% ethanol. The sections were proceeded for antigen retrieval by a standard microwave heating technique either using diluted 50x Envision FLEX Target Retrieval Buffer (pH9, #K8004, DAKO) for ANXA1, CD68, CD86, CD206/MRC1, CD4, CD8α or diluted 10x AR6 sodium citrate buffer (pH6, #AR600250ML, Akoya BioSciences) for S100A6, p-STAT3 (Tyr705). The sections were immersed in blocking/antibody diluent (ARD1001EA, Akoya Biosciences) for 10 minutes at room temperature and were subsequently incubated with primary antibodies (ANXA1, 1:2000, #ab214486, Abcam; CD68, CD86, CD206/MRC1, CD4, CD8α: 1:500, #97778S, #19589S, #24595T, #25229S, #98941S, Cell Signalling Technology; S100A6, 1:500, #10245-1-AP, Proteintech; p-STAT3 (Tyr705), 1:100, #9131S, Cell Signalling Technology). The sections were then washed thoroughly with 1X TBST and incubated with Opal polymer HRP Mouse+Rabbit (#ARH1001EA, Akoya Biosciences) for 30 minutes at room temperature. After a brief wash with 1X TBST, Opal fluorophore (1:100) was applied for S100A6, CD68, and CD4 (Opal 570), p-STAT3 (Tyr705), CD206/MRC1, and CD8α (Opal 520), ANXA1 and CD86 (Opal 690) for 15 minutes at room temperature. The section slides were counterstained with DAPI solution (1:1000, Akoya) and mounted for examination by Nikon Eclipse Ti2-E Live-cell Fluorescence Imaging System

**Tissue dissociation and preparation of live single-cell suspension**

The established LenR PY003 PDTX HCC tumor was isolated from NOD/SCID mice. The tumor sample was first cropped into small pieces and were transferred to gentleMACS C tubes (Miltenyi Biotec) supplemented with 0.1 g/ml Collagenase IV and 10 mg/ml DNaseI, and then were dissociated in dissociation buffer using gentleMACS dissociator (Miltenyi Biotec) according to the manufacturer’s instructions. The resulting single-cell suspension was filtered through sterile 70 μm cell strainers (SPL Life Sciences). Lastly, the cells were sorted by BD FACSAria III Cell Sorter using LIVE/DEAD^TM^ Fixable Near-IR Dead Cell Stain Kit (#L34975, Invitrogen, Thermo Fisher Scientific).

**Single-cell RNA sequencing (scRNA-seq)**

Live cells dissociated from HCC tumor bulk stored in ice-cold FACS buffer were immediately processed according to manufacturer’s guidelines. This involved encapsulating the cells into single-cell gel beads (GEMs) and applying unique molecular identifier (UMI) barcoding using the Chromium Next GEM 3’ v3 chemistry Single-Cell kit (10X Genomics) at The Beijing Genomics Institute. Libraries were then prepared and sequenced on a NovaSeq platform (Illumina, San Diego, USA). The scRNA sequencing data generated by this study have been deposited to GEO database under accession number: GSE302059.

**Processing and quality control of scRNA sequencing data**

Initial data demultiplexing, read alignment, UMI counting, and annotation of the raw read data were performed using the Cell Ranger Single-Cell software version 5.0.1 (10X Genomics). Raw sequencing reads of the two samples (LenR and Mock) were mapped to the human genome (GRCh38) and mouse genome (mm10) and were then analyzed with the Seurat R package version 4.1.1. Datasets of two samples (LenR and Mock) were merged as one Seurat object before quality control. The cells were filtered based on the following criteria: 500 < nFeature < 6000, nCount < 25000, mitochondria < 20%, and hemoglobin < 1%. The gene expression data was normalized and variance-stabilized using SCTransform function.

**Animals**

All mice were housed in university facility in 12 hours light/dark cycle (8:00-20:00 light, 20:00-8:00 dark) with controlled room temperature (23±2°C) and humidity (30-70%), in groups according to stocking density as recommended. The study protocol received approval and was conducted in line with the guidelines for the Use of Live Animals in Teaching and Research at Hong Kong Polytechnic University. In the subcutaneous tumor model, the tumor volumes were kept within 10% of normal body weight or 1.3 cm in diameter. Mice were sacrificed if they experienced more than a 20% loss in body weight. In the HTVI model, mice were euthanized if their body weight increased by more than 10% or decreased by over 20%. The maximum allowable tumor size and burden adhered to the study protocol of Hong Kong Polytechnic University, and this limit was not exceeded in any of the experiments.

***In vivo* tumorigenicity assay**

Human HCC cells were suspended in DMEM and Matrigel Matrix (BD Biosciences) with a 1:1 ratio and subcutaneously injected into the flanks of male NOD/SCID mice of age 4-6-week-old. The developed tumors were harvested at the end of the experiment. Tumor-initiating cell frequency was calculated using ELDA software^1^.

**Establishment of LenR PDTX HCC mouse model**

HCC tumor sample was obtained from PY003 patient who underwent hepatectomy. The patient did not receive any previous local or systemic treatment prior to the surgery. The patient tumor sample was dissociated and then transplanted into immunodeficient NOD/SCID mice. After tumor implantation, the mice were orally administered four rounds of 30 mg/kg/day lenvatinib. The tumor was transferred to the secondary, tertiary, and quaternary mouse recipients to develop LenR PDTX HCC tumors. The first three rounds were 31 days each and the fourth round was 16 days due to the large tumor size. The control counterparts were administered with PBS.

**Establishment of LenR RIL175 HCC mouse model**

RIL175 cells were orthotopically injected to 6- to 8-week-old male wild-type C57BL/6J mouse liver. The mice were then orally administrated with 30 mg/kg/day lenvatinib for 25 days. The tumor growth was monitored by *in vivo* imaging system (IVIS) (Perkin-Elmer). Successful establishment of lenvatinib resistance HCC was evaluated by the IVIS.

**Establishment of LenR *Tp53*^KO^/*Myc*^OE^ HCC mouse model**

Plasmids encoding *MYC* (15 μg), *sgTp53* (15 μg), and sleeping beauty transposase (2.5 μg) were diluted in 2 mL of saline (0.9% NaCl), filtered through a 0.22 μm filter, and injected into 6- to 8-week-old male wild-type C57BL/6J mice via HTVI. After 12 days post injection, mice were orally administrated with 30 mg/kg/day lenvatinib for 25 days. The tumor growth was monitored by IVIS. Successful establishment of lenvatinib resistance HCC was evaluated by the IVIS.

**Knockdown of Anxa1 in LenR *Tp53*^KO^/*Myc*^OE^ HCC mouse model**

After establishing LenR *Tp53*^KO^/*Myc*^OE^ HCC tumors, all mice were randomly assigned to respective groups to ensure similar initial bioluminescent signal intensity of liver tumors before administration of treatment between groups. The mice were intravenously injected with AAV8-*shAnxa1* virus or AAV8-NTC as control (1x10^12^vg/mouse). After the viral injection, the mice were administrated with 30 mg/kg lenvatinib or saline daily. The tumor growth was monitored by IVIS.

**Supplementary Fig. 1. Identification of ANXA1 overexpression in LenR HCC.** **A** Feature plot of marker gene expression for HCC (*ALB* and *KRT8*) and immune cells (*PTPRC*). ALB: albumin; KRT8: keratin 8; PTPRC: protein tyrosine phosphatase receptor type c. **B** *ANXA1* mRNA expression of LenR and Mock PDTX tumors (n=3 independent experiments, two-tailed *t* test). **C** *ANXA1* mRNA expression in LenR HCC cell lines (n=3 independent experiments, two-tailed *t* test). **D** ANXA1 protein expression in LenR HCC cell lines and their mock controls (n=5 independent experiments, two-tailed *t* test). **E** IHC staining of ANXA1 in LenR PLC/PRF/5 cell-derived tumors and mock controls. Five images were randomly taken for each group. ANXA1 expression was quantified by FIJI (ImageJ) (n=5, two-tailed *t* test). Data was presented as mean ± standard deviation. **p* < 0.05, ***p*<0.01 & *****p* < 0.0001.

**Supplementary Fig. 2. Identification of ANXA1 overexpression in two LenR HCC mouse models.** **A-B** IHC staining analysis of ANXA1 expression in LenR *Tp53*^KO^/*Myc*^OE^ HCC and LenR RIL175 HCC mouse tumor tissues and their corresponding mock controls. Five images were randomly taken for each sample group (n=5, two-tailed *t* test). **C** IHC staining analysis of ANXA1 expression in non-tumor liver tissues and *Tp53*^KO^/*Myc*^OE^-derived HCC mouse tumor tissues without lenvatinib and with 8, 14, 19, 21, and 37 days lenvatinib treatment. Five images were randomly taken for each sample group. Bar plot showed ANXA1 protein expression in each sample group (n=5, two-tailed *t* test). Data was presented as mean ± standard deviation. **p* < 0.05, ***p* < 0.01 & ****p* < 0.001.

**Supplementary Fig. 3. Positive correlation between ANXA1 expression and cancer stemness. A** UMAP plot showing the distribution of *ANXA1*^High^ and *ANXA1*^Low^ groups based on *ANXA1* mean expression in LenR HCC and Mock HCC cell populations by using our scRNA-seq data **B** Dot plot of the expression of CSC markers (*CD24*, *CD47*, *BMI1*, *KLF4*, *PROM1*) in the *ANXA1*^High^ and *ANXA1*^Low^ groups in LenR HCC cell population. CD24: Cluster of differentiation 24; CD47: Cluster of Differentiation 47; BMI1: B cell-specific moloney murine leukemia virus integration site 1; KLF4: Krüppel-like factor 4; PROM1: prominin 1. **C** Positive correlation between *ANXA1* expression and CSC markers (*CD24*, *CD47*, *BMI1*, *KLF4*, *PROM1*) in the TCGA-LIHC patient cohort.

**Supplementary Fig. 4.** **Verification of successful ANXA1 knockdown. A** Quantification of western blot analyses of ANXA1 expression upon knockdown of ANXA1 in PLC/PRF/5 and Huh7 (n=3 independent experiments, one way ANOVA followed by Tukey’s multiple comparison test). **B** Immunofluorescent staining of ANXA1 expression (green) in sh*ANXA1* PLC/PRF/5 and Huh7. DAPI (blue) denoted nuclear staining. **C** IHC staining of ANXA1 expression in tumor xenografts. Five images were randomly taken for each sample group. ANXA1 protein expression was quantified by FIJI (ImageJ) (n=5, two-tailed *t* test). Scale bar: 5 µm. **D** QPCR analyses of differentiation markers *SERPINA1* and *HNF4A* in sh*ANXA1* PLC/PRF/5 and Huh7 (n=4 independent experiments, one way ANOVA followed by Tukey’s multiple comparison test). Data was presented as mean ± standard deviation. **p*<0.05, ***p*<0.01, ****p* < 0.001 &*****p* < 0.0001.

**Supplementary Fig. 5. Overexpression of ANXA1 induces cancer stemness and lenvatinib resistance.** **A** Verification of successful ANXA1 overexpression by Western blot (n=6 independent experiments, two tailed t test). **B** CSC frequency of each group was calculated based on number of formed tumor spheres derived from EV or OE ANXA1 Huh7 (n=2 independent experiments, one-sided extreme limiting dilution with chi-square analysis). **C** Representative photos of tumors derived from EV or OE ANXA1 Huh7 with tumor incidence rate being indicated. **D** Representative contour diagrams illustrating the percentage of annexin V-positive EV and OE ANXA1 Huh7 treated with 40 µM lenvatinib treatment or DMSO. Bar plot showing the percentage of apoptotic cells after 40 µM lenvatinib treatment or DMSO (n=3 independent experiments, two-tailed *t* test). EV: empty vector, OE: overexpression. Data was presented as mean ± standard deviation. **p* < 0.05 & ***p<0.001.

**Supplementary Fig. 6. Secretory ANXA1 induces lenvatinib resistance and tumor sphere formation *in vitro*. A** Representative contour diagrams illustrating the percentage of annexin V-positive Huh7 cells incubated with conditioned media (CM) derived from EV or OE ANXA1 cells treated with 40 µM lenvatinib treatment or 40 µM DMSO as control. Bar plot showing the percentage of apoptotic cells after 40 µM lenvatinib treatment or DMSO (n=3 independent experiments, two-tailed *t* test). **B** CSC frequency of each group was calculated based on number of formed tumor spheres derived from Huh7 cells incubated with conditioned media derived from EV or OE ANXA1 cells (n=2 independent experiments, one-sided extreme limiting dilution analysis with chi-square test). Data was presented as mean ± standard deviation. **p* < 0.05.

**Supplementary Fig. 7.** **Identification of S100A6 overexpression in LenR HCC. A** *ANXA1* and *S100A6* expression (left) and their correlations with tumor stage (right) in the [TCGA-LIHC](https://portal.gdc.cancer.gov/projects/TCGA-LIHC) dataset. **B** *S100A6* mRNA expression fold change in LenR HCC cell lines PLC/PRF/5, Huh7, and MHCC-97L relative to the corresponding mock controls (n=3 independent experiments, two-tailed *t* test). **C** S100A6 protein expression in LenR HCC cell lines and their mock controls (n=3 independent experiments, two-tailed t test). **D** IHC staining of S100A6 expression in tumor tissue samples derived from LenR and Mock PLC/PRF/5. Five images were randomly taken for each sample group. Bar plot showed S100A6 protein expression in each sample group (n=5, two-tailed *t* test). Data was presented as mean ± standard deviation. **p* < 0.05, ***p*<0.01 & *****p* < 0.0001.

**Supplementary Fig. 8. Positive correlation between S100A6 expression and cancer stemness. A** UMAP plot showing distribution of *S100A6*^High^ and *S100A6*^Low^ groups based on *S100A6* mean expression in LenR and Mock HCC cell populations by using our scRNA-seq data. **B** Expression of CSC markers in *S100A6*^High^ and *S100A6*^Low^ groups. **C** Positive correlation between *S100A6* and CSC markers in TCGA-LIHC dataset. SOX9: SRY-Box transcription factor 9; EPCAM: epithelial cell adhesion molecule. **D** UMAP plot illustrating high and low stemness clusters in LenR HCC cell population by cellular (cyto) trajectory reconstruction analysis using gene counts and expression (CytoTRACE) analysis. Bar plot showing *S100A6* expression high stemness compared to low stemness clusters in LenR HCC. **E**. β-catenin expression in Mock NTC, LenR NTC, LenR sh*ANXA1*#1 EV, and LenR sh*ANXA1*#1 OE S100A6 Huh7 (n=3 independent experiments, one way ANOVA followed by Tukey’s multiple comparison test). Data was presented as mean ± standard deviation. ***p*<0.01 & *****p* < 0.0001.

**Supplementary Fig. 9**. **Regulatory role of ANXA1 on IL6/JAK2 pathway**. **A** Spearman correlation analysis between *ANXA1* and *IL6* mRNA expression levels in HCC based on the TCGA-LIHC dataset. **B** Western blot showing alterations of pJAK2 (Tyr1007/1008) expression upon suppression or overexpression of ANXA1 (n=3 independent experiments, one way ANOVA followed by Tukey’s multiple comparisions test for sh*ANXA1* data while two-tailed *t* test for overexpression data). Data was presented as mean ± standard deviation. * *p*<0.05, ***p* < 0.01 & *****p* < 0.0001.

**Supplementary Fig. 10**. **ANXA1 regulates S100A6 expression via activation of STAT3 signaling pathway. A** pSTAT3 inhibitor S3I-201 suppressed the expression of pSTAT3 (Tyr705), SOX2 and S100A6 in ANXA1-overexpressing cells (n=5 independent experiments, one way ANOVA followed by Tukey’s multiple comparison analysis). **B** Venn diagram showing the intersection of predicted transcription factors binding to S100A6 promoter region across TRANSFAC, GTRD and JASPAR, highlighting STAT3 as one of the 18 common consensus factors. **C** Consensus STAT3 DNA-binding motif retrieved from JASPAR. **D** Schematic diagram illustrating three predicted STAT3 binding sites located within S100A6 promoter region. **E** S3I-201 at 100µM sensitized LenR and ANXA1-overexpressing cells to lenvatinib treatment at 40µM for 48 hours (n=3 independent experiments, one way ANOVA followed by Tukey’s multiple comparison analysis). **p*<0.05; ***p*<0.01, ****p*<0.001 & *****p*<0.0001.

**Supplementary Fig. 11. Identification of SOX2 overexpression in LenR HCC. A** *SOX2* mRNA expression fold change in LenR cells relative to the corresponding mock controls (n=3 independent experiments, two-tailed *t* test). **B** Quantification of western blot analyses of SOX2 expression in mock and lenR HCC cells (n=4 independent experiments, two-tailed *t* test). **C** IHC staining of SOX2 expression in tumor tissue samples derived from LenR and Mock PLC/PRF/5 cells. Five images were randomly taken for each sample group. SOX2 protein expression was quantified by FIJI (ImageJ). Bar plot showed SOX2 protein expression (n=5, two-tailed *t* test). **D** Correlation analysis between *SOX2* and *ANXA1* expression in TCGA-LIHC dataset for HCC patients. **E** Quantification of western blot anlayses of SOX2 expressin in Huh7 LenR shSOX2 cells (n=4 independent experiments, one way ANOVA followed by Tukey’s multiple comparisions test). Data was presented as mean ± standard deviation. * *p*<0.05, ***p* < 0.01 & ****p* < 0.001.

**Supplementary Fig. 12. STAT3 regulates SOX2 expression. A** Schematic diagram illustrating the prediction of STAT3 binding to SOX2 promoter by TF-binding site database GTRD. **B** Consensus binding motif of STAT3. **C** Correlation analysis between *SOX2* and *STAT3* expression. **D** Western blot showing p-STAT3 (Tyr705), total STAT3, SOX2 protein expression in LenR and Mock Huh7 cells treated with DMSO or 100 µM S3I-201 (n=3 independent experiments, one way ANOVA followed by Tukey’s multiple analysis). **e** Western blot showing the suppression of SOX2 expression upon ANXA1 knockdown (n=3 independent experiments, one way ANOVA followed by Tukey’s multiple analysis). **p*<0.05, ***p*<0.01; ****p*<0.001 & *****p*<0.0001.

**Supplementary Fig. 13. Identification of cell types in mouse cell population by scRNA-seq analysis. A** Dot plot of marker gene expression for NK cells (*Nkg7*, *Ncr1*, Trdc), macrophages (C1qc, Trem2, Ms4a7), endothelial cells (Eng, Flt1, Cdh5) and monocytes (*Ly6c2*, *Ccr2*, *Jaml*). Nkg7: natural killer cell granule protein 7; Ncr1: natural cytotoxicity triggering receptor 1; Trdc: t cell receptor delta constant; C1qc: complement C1q C chain; Trem2: triggering receptor expressed on myeloid cells 2; Ms4a7: membrane-spanning 4-domains subfamily A member 7; Eng: endoglin; Flt1: vascular endothelial growth factor receptor 1; Cdh5: cadherin 5; Ly6c2: lymphocyte antigen 6C2; Ccr2: CC-chemokine receptor-2; Jaml: Junction Adhesion Molecule Like. **B** Dot plot of marker gene expression of M2 macrophages (*Arg1*, *Cd36*, *Spp1*) and M1 macrophages (*Irf1*, *Stat1*, *Ccl4*). Arg1: ARG1 arginase 1; Spp1: secreted phosphoprotein 1; Irf1: interferon regulatory factor 1; Stat1: signal transducer and activator of transcription 1; Ccl4: CC Motif Chemokine Ligand 4. **C** UMAP plot showing the distribution of M1 and M2 macrophages in mouse LenR and Mock cell populations.

**Supplementary Fig. 14. Increased infiltration of M2 macrophages in LenR HCC TME in three LenR HCC mouse models. A-C** IHC staining analysis of CD163 expression in three different LenR HCC mouse models (LenR PDTX, LenR *Tp53*^KO^/*Myc*^OE^ and LenR RIL175). Five images were randomly taken for each sample group. CD163 protein expression was quantified by FIJI (ImageJ). Bar plot showed CD163 protein expression in each sample group (n=5, two-tailed *t* test). **D** Correlation analysis between *ANXA1* expression and M2 macrophage marker expression of *CD163*, *MRC1 (CD206)*, *CD36*. CD163: cluster of differentiation 163; MRC1: mannose receptor C-type 1; CD36: cluster of differentiation 36. **E** Correlation analysis between *ANXA1* expression and M2 macrophage infiltration by cell-type identification by estimating relative subsets of RNA transcripts (CIBERSORT). Data was presented as mean ± standard deviation. ***p* < 0.01 & *****p* < 0.0001.

**Supplementary Fig. 15. Identification of the role of tumor-derived ANXA1 in macrophage polarization *in vitro*.** **A** Secretory ANXA1 concentration from Mock NTC, LenR NTC, LenR sh*ANXA1*#1, and LenR sh*ANXA1*#2 PLC/PRF/5 and Huh7 cells detected by ELISA assay (n=3 independent experiments, two-tailed *t* test). **B** Percentage of CD86^+^ cells among differentiated THP-1 after incubating with CM derived from respective groups (n=3 independent experiments, two-tailed *t* test). CD86: cluster of differentiation 86. **C** Quantification of western blot analyses of p-ERK(Thr202/Tyr204) and p-NFκB p65 (Ser536) (n=5 independent experiments, one way ANOVA followed by Tukey’s multiple comparison analysis). **D** Schematic diagram illustrating the role of N-terminal ANXA1 interacting with FPR2 in inducing intracellular ERK and NF-κB signalling activation of THP-1 derived macrophages, thereby driving M2 macrophage polarization. Data was presented as mean ± standard deviation. **p* < 0.05, ***p* < 0.01 & ****p*<0.001.

**Supplementary Fig. 16. Verification of the intrinsic Anxa1-mediated signalling pathway via Anxa1 knockdown *in vivo.*** Multiplex staining of ANXA1, p-STAT3, and S100A6 expression in NTC-Lenvatinib, sh*Anxa1*-Lenvatinib, and sh*Anxa1*-Vehicle tumor tissue samples. DAPI denoted nuclear staining. Scale bar: 200 µm.

**Supplementary Fig. 17. The alteration of M1/M2 macrophage and CD4/CD8 T cell composition post Anxa1 knockdown treatment *in vivo*.** **A-B** Multiplex staining of CD68, CD206, CD86, CD4, and CD8 expression in NTC-Lenvatinib, sh*Anxa1*-Lenvatinib, and sh*Anxa1*-Vehicle tumor tissue samples. DAPI denoted nuclear staining. Scale bar: 200 µm.

**Supplementary Fig. 18.** Schematic diagram illustrating the intrinsic role of ANXA1 in promoting cancer stemness via SOX2/STAT3/S100A6 feedback loop and the extrinsic role of ANXA1 in driving M2 macrophage polarization and building up an immunosuppressive TME, thereby inducing lenvatinib resistance in HCC.

**Supplementary Tables**

**Supplementary Table 1.** Clinicopathological features of lenvatinib-sensitive and lenvatinib-resistant HCC patients.

| **Patient number** | **Lenvatinib response** | **Sex** | **Age** | **HBV status** | **Tumor Size** | **No. of nodules** | **No. of lymph node metastasis** | **Presence of venous infiltration** | **Presence of extrahepatic metastasis** |
| --- | --- | --- | --- | --- | --- | --- | --- | --- | --- |
| #1 | Resistant | Male | 43 | +ve | 84×71mm | 1 | 2 | Yes | No |
| #2 | Resistant | Male | 51 | neg | 60×56mm | 3+ | 0 | No | No |
| #3 | Sensitive | Male | 57 | +ve | 118×76mm | 1 | 0 | No | No |
| #4 | Sensitive | Male | 55 | +ve | 148×112mm | 1 | 0 | No | No |

**Supplementary Table 2**. **Effect of ANXA1 knockdown and overexpression on tumorigenicity of PLC/PRF/5 and Huh7 cells. a-b** Subcutaneous *in vivo* tumor development in NOD/SCID mice of Mock NTC, LenR NTC, LenR sh*ANXA1*#1, LenR sh*ANXA1*#2 PLC/PRF/5 and Huh7 cells. **c** Subcutaneous *in vivo* tumor development in NOD/SCID mice of EV and OE ANXA1 Huh7 cells.

**A** Primary engraftment of PLC/PRF/5 cells with ANXA1 knockdown

| PLC/PRF/5 | Tumor incidence rate | | | Extreme limiting dilution | | |
| --- | --- | --- | --- | --- | --- | --- |
|  | 5x10^2^  cells | 5x10^3^ cells | 1x10^4^  cells | Estimated CSC frequency | 95% CI | *p*-value |
| Mock NTC | 2/5 | 2/5 | 2/5 | 1/9990 | 1/23829-1/4189 | 3.46e-5 |
| LenR NTC | 3/5 | 5/5 | 5/5 | 1/545 | 1/1742-1/171 | / |
| LenR sh*ANXA1*#1 | 2/5 | 2/5 | 2/5 | 1/9990 | 1/23829-1/4189 | 3.46e-5 |
| LenR sh*ANXA1*#2 | 0/5 | 3/5 | 3/5 | 1/8571 | 1/19754-1/3719 | 0.000113 |

**B** Primary engraftment of Huh7 cells with ANXA1 knockdown

| Huh7 | Tumor incidence rate | | | Extreme limiting dilution | | |
| --- | --- | --- | --- | --- | --- | --- |
|  | 5x10^2^  cells | 5x10^3^ cells | 1x10^4^  cells | Estimated CSC frequency | 95% CI | *p*-value |
| Mock NTC | 1/5 | 3/5 | 2/5 | 1/9564 | 1/22577-1/4052 | 2.1e-8 |
| LenR NTC | 5/5 | 5/5 | 5/5 | 1/1 | 1/627 – 1/1 | / |
| LenR sh*ANXA1*#1 | 2/5 | 3/5 | 4/5 | 1/4603 | 1/9899-1/2141 | 2.96e-6 |
| LenR sh*ANXA1*#2 | 1/5 | 3/5 | 2/5 | 1/9564 | 1/22577-1/4052 | 2.1e-8 |

**C** Primary engraftment of Huh7 cells with ANXA1 overexpression

| Huh7 | Tumor incidence rate | | | Extreme limiting dilution | | |
| --- | --- | --- | --- | --- | --- | --- |
|  | 5x10^2^  cells | 1x10^3^ cells | 5x10^3^  cells | Estimated CSC frequency | 95% CI | *p*-value |
| EV | 2/5 | 4/5 | 1/5 | 1/3840 | 1/9293-1/1587 | / |
| OE ANXA1 | 2/5 | 5/5 | 5/5 | 1/525 | 1/1163-1/237 | 0.000515 |

**Supplementary Table 3. shRNA sequences for gene knockdown.**

|  | **Sequence (5'-3')** |
| --- | --- |
| **NTC** | CCGGTTGTGCTCTTCATCTTGTTGCCGGCAACAAGATGAAGAGCACCAATTTTTG |
| **sh*ANXA1*#1** | GCTATGATCAGAAGACTTTAAT |
| **sh*ANXA1*#2** | GAGATCTGGCCAAAGACATAA |
| **sh*SOX2*#1** | CTGCCGAGAATCCATGTATAT |
| **sh*SOX2*#2** | GTACAGTATTTATCGAGATAA |

**Supplementary Table 4. Primer sequences that target two predicted SOX2 binding sites in the ANXA1 promoter region for qPCR.**

|  | **Forward primer (5’-3’)** | **Reverse primer (5’-3’)** |
| --- | --- | --- |
| **Site 1** | AATTATTCGGCACTGTGTAATGTT | CTAGAATTCTTGTGACAATGGAGAA |
| **Site 2** | ACTGGTATTAGGATTGGGGCA | GTTTGAAGTACATGGCTCAGCT |

**Supplementary Table 5. List of sequences of FL or truncated ANXA1 promoter.**

FL ANXA1 promoter (1kb): GTATCAAAATGTATATATAACTGATCTATATTACAGTTTATATATATAGTTCTATGTAACTGACTCTATTTAAAAGAATTAATGTAGATAGGCCTTCTGATGTCAATGCTTTTATTGAATGTCATAATGATCAAATTTTTGGCATTACCTTTGTTCAAAATTTTGAGCCAATCTGGAACTTTATTACTTCTATATTTCTTATTTATAACAACAAGCTATATCTAGGAACAAAAGTCGTTTATTTTTTAAGGCAAGAAAAATATAAATAGAAAATTATTCGGCACTGTGTAATGTTAAAATTTACAAATATATGTTATTTCTCCATTGTCACAAGAATTCTAGTGGGTAGACAACTTAGATAATGCTCCTATATACAAAAGTAAAAACAGCTAGGCATGGTGGCTCACGCCTGTGATTCCAACTCTTAGGGAGACAGAGGCAGGAGGATAGCCTGAGCCCAGGAGTTTGAGACCTGCCTGGGCAATATAACGAGACCCCATTCTCCACAAAAAGCGGGGGAAAAAGACAACAAAACAAAAGTAAAAACAGGTTCAGAAAAATTAAGTATGTCCCTAAAGTCACAGAATAATAAGTGATGGAGGTGGAATTCAAATGTAATATTTTGTTTGTTGGTTTGTTTAGTTTTGAAGGTAACATACTTTTTAGTCTACAACCTATTTAACTACAAAAGTGTGCCAATCACTGGTATTAGGATTGGGGCAGATGTATACTCTGAAAATAAAAGTGCAAAATAAATATCCCATCTGGAAATAAAAGTGCAAAATAAAGAGGGAGGAAAAATCAGCCCTTCCATTTCTTTTCCTTTGTAATGCCAGTTGAATTGGTTTAGTGAGTAGTTTTGCAAGACTTGTTTCACTTTGTTTTTGGACATAGCTGAGCCATGTACTTCAAACAGAAGGCAGCCAATTACTAACTTCTGGTTGCTAGGTGTGGCTTCCTTTAAAATCCTATAAAATCAGAAGCCCAAGTCTCCACTGCC

pT1 truncated ANXA1 promoter (778bp): GTATCAAAATGTATATATAACTGATCTATATTACAGTTTATATATATAGTTCTATGTAACTGACTCTATTTAAAAGAATTAATGTAGATAGGCCTTCTGATGTCAATGCTTTTATTGAATGTCATAATGATCAAATTTTTGGCATTACCTTTGTTCAAAATTTTGAGCCAATCTGGAACTTTATTACTTCTATATTTCTTATTTATAACAACAAGCTATATCTAGGAACAAAAGTCGTTTATTTTTTAAGGCAAGAAAAATATAAATAGAAAATTATTCGGCACTGTGTAATGTTAAAATTTACAAATATATGTTATTTCTCCATTGTCACAAGAATTCTAGTGGGTAGACAACTTAGATAATGCTCCTATATACAAAAGTAAAAACAGCTAGGCATGGTGGCTCACGCCTGTGATTCCAACTCTTAGGGAGACAGAGGCAGGAGGATAGCCTGAGCCCAGGAGTTTGAGACCTGCCTGGGCAATATAACGAGACCCCATTCTCCACAAAAAGCGGGGGAAAAAGACAACAAAACAAAAGTAAAAACAGGTTCAGAAAAATTAAGTATGTCCCTAAAGTCACAGAATAATAAGTGATGGAGGTGGAATTCAAATGTAATATTTTGTTTGTTGGTTTGTTTAGTTTTGAAGGTAACATACTTTTTAGTCTACAACCTATTTAACTACAAAAGTGTGCCAATCACTGGTATTAGGATTGGGGCAGATGTATACTCTGAAAATAAAAGTGCAAAATAAATATCCCATCTGGAAATAAAAGT

pT2 truncated ANXA1 promoter (222bp):

GCAAAATAAAGAGGGAGGAAAAATCAGCCCTTCCATTTCTTTTCCTTTGTAATGCCAGTTGAATTGGTTTAGTGAGTAGTTTTGCAAGACTTGTTTCACTTTGTTTTTGGACATAGCTGAGCCATGTACTTCAAACAGAAGGCAGCCAATTACTAACTTCTGGTTGCTAGGTGTGGCTTCCTTTAAAATCCTATAAAATCAGAAGCCCAAGTCTCCACTGCC

pT3 truncated ANXA1 promoter (107bp):

GCTGAGCCATGTACTTCAAACAGAAGGCAGCCAATTACTAACTTCTGGTTGCTAGGTGTGGCTTCCTTTAAAATCCTATAAAATCAGAAGCCCAAGTCTCCACTGCC

**Supplementary Table 6. Primer sequences for RT-qPCR.**

| **Gene** | **Forward primer (5’-3’)** | **Reverse primer (5’-3’)** |
| --- | --- | --- |
| ***ANXA1*** | AGCGTCAACAGATCAAAGCAGCAT | AGACCCTGTTAATGTCTCTGATTT |
| ***S100A6*** | GTGACAAGCACACCCTGAGCAA | GGAAGTTCACCTCCTGGTCCTT |
| ***SOX2*** | ATGCACCGCTACGACGTGA | CTTTTGCACCCCTCCCATTT |
| ***CD163*** | TTTGTCAACTTGAGTCCCTTCAC | TCCCGCTACACTTGTTTTCAC |
| ***MRC1* (CD206)** | GGGTTGCTATCACTCTCTATGC | TTTCTTGTCTGTTGCCGTAGTT |
| ***SERPINA1*** | ATGCTGCCCAGAAGACAGATA | CTGAAGGCGAACTCAGCCA |
| ***HNF4A*** | CCACGGGCAAACACTACGG | GGCAGGCTGCTGTCCTCAT |
| ***HPRT*** | CCTGGCGTCGTGATTAGTGAT | AGACGTTCAGTCCTGTCCATAA |

**References**

1	Hu, Y. & Smyth, G. K. ELDA: extreme limiting dilution analysis for comparing depleted and enriched populations in stem cell and other assays. *J Immunol Methods* **347**, 70-78 (2009). <https://doi.org/10.1016/j.jim.2009.06.008>
